# Mentoring in palliative medicine in the time of covid-19: a systematic scoping review: Mentoring programs during COVID-19

**DOI:** 10.1186/s12909-022-03409-4

**Published:** 2022-05-11

**Authors:** Sherill Goh, Ruth Si Man Wong, Elaine Li Ying Quah, Keith Zi Yuan Chua, Wei Qiang Lim, Aubrey Ding Rui Ng, Xiu Hui Tan, Cheryl Shumin Kow, Yao Hao Teo, Elijah Gin Lim, Anushka Pisupati, Eleanor Jia Xin Chong, Nur Haidah Ahmad Kamal, Lorraine Hui En Tan, Kuang Teck Tay, Yun Ting Ong, Min Chiam, Alexia Sze Inn Lee, Annelissa Mien Chew Chin, Stephen Mason, Lalit Kumar Radha Krishna

**Affiliations:** 1 Yong Loo Lin School of Medicine, National University of Singapore, NUHS Tower Block, 1E Kent Ridge Road, Level 11, Singapore, 119228 Singapore; 2 Division of Supportive and Palliative Care, National Cancer Centre Singapore, 11 Hospital Cr, Singapore, 169610 Singapore; 3 Division of Cancer Education, National Cancer Centre Singapore, 11 Hospital Cr, Singapore, 169610 Singapore; 4 Medical Library, National University of Singapore Libraries, Block MD6, Centre for Translational Medicine, 14 Medical Drive, #05-01, Singapore, 117599 Singapore; 5 Palliative Care Institute Liverpool, Academic Palliative & End of Life Care Centre, University of Liverpool, Cancer Research Centre, University of Liverpool, 200 London Rd, Liverpool, L3 9TA UK; 6 Duke-NUS Medical School, National University of Singapore, 8 College Rd, Singapore, 169857 Singapore; 7 Centre of Biomedical Ethics, National University of Singapore, 21 Lower Kent Ridge Rd, Singapore, 119077 Singapore; 8 PalC, The Palliative Care Centre for Excellence in Research and Education, PalC c/o Dover Park Hospice, 10 Jalan Tan Tock Seng, Singapore, 308436 Singapore

**Keywords:** Mentoring, Interprofessional Mentoring, E-mentoring, Peer Mentoring, Near-peer mentoring, Palliative Medicine

## Abstract

**Introduction:**

The redeployment of mentors and restrictions on in-person face-to-face mentoring meetings during the COVID-19 pandemic has compromised mentoring efforts in Palliative Medicine (PM). Seeking to address these gaps, we evaluate the notion of a combined novice, peer-, near-peer and e-mentoring (CNEP) and interprofessional team-based mentoring (IPT) program.

**Methods:**

A Systematic Evidence Based Approach (SEBA) guided systematic scoping review was carried out to study accounts of CNEP and IPT from articles published between 1st January 2000 and 28th February 2021. To enhance trustworthiness, concurrent thematic and content analysis of articles identified from structured database search using terms relating to interprofessional, virtual and peer or near-peer mentoring in medical education were employed to bring together the key elements within included articles.

**Results:**

Fifteen thousand one hundred twenty one abstracts were reviewed, 557 full text articles were evaluated, and 92 articles were included. Four themes and categories were identified and combined using the SEBA’s Jigsaw and Funnelling Process to reveal 4 domains - characteristics, mentoring stages, assessment methods, and host organizations. These domains suggest that CNEP’s structured virtual and near-peer mentoring process complement IPT’s accessible and non-hierarchical approach under the oversight of the host organizations to create a robust mentoring program.

**Conclusion:**

This systematic scoping review forwards an evidence-based framework to guide a CNEP-IPT program. At the same time, more research into the training and assessment methods of mentors, near peers and mentees, the dynamics of mentoring interactions and the longitudinal support of the mentoring relationships and programs should be carried out.

**Supplementary Information:**

The online version contains supplementary material available at 10.1186/s12909-022-03409-4.

## Introduction

Mentoring in Palliative Medicine (PM) [1] has been shown to boost a physician’s career and personal development [2, 3], enhance collaborations [4, 5], and advance the academic standing of the host organisations overseeing mentoring programs [6]. It also shapes a mentee’s “*conceptual model from disease and diagnosis to patient goals, prognosis and function*”, reinforcing attention upon improving patient care and quality of life [7]. Built upon *“personalised and enduring mutually beneficial relationships between an experienced clinician, junior clinicians and/or undergraduates and the host organization”* [8], novice mentoring, which is the dominant mentoring approach in PM has been especially compromised by COVID-19 restrictions [9–11] including the re-deployment of mentors to the ‘frontlines’ and restrictions on in-person meetings [12]. These limitations have compromised mentoring support [13] and raised the risk of inadequate oversight [14] of mentoring relationships and assessments of progress, potentiating the danger of ethical, legal and professional lapses in mentoring (henceforth ethical issues in mentoring) [15].

Although supplementing novice mentoring with peer [16] and electronic mentoring (e-mentoring), also known as CNEP mentoring (henceforth CNEP) [17], circumvents restrictions on face-to-face meetings, improves timely and holistic support [18] and fosters high quality mentoring relationships [19, 20], problems persist. Therefore, we evaluate the possibility of further supplementing CNEP with mentoring support from senior members of PM’s interprofessional teams (IPT) [21–24]. Consisting of physicians, nurses, medical social workers, physiotherapists and/or occupational therapists, IPT-based mentoring (henceforth IPT) allows senior healthcare professionals within interprofessional teams [24] to step up to fulfil the usual mentoring role of the senior physician [25]. The use of IPT is further strengthened by evidence that mentoring in nursing [26], medical social work [27], physiotherapy and occupational therapy [28] shares significant commonalities with novice mentoring in medicine [29].

### Need for this review

It is with this impetus to address the prevailing threats to novice mentoring [30] and a general lack of data on the use of CNEP and IPT that, a systematic scoping review (SSR) is proposed. The data accrued provides a means of designing and evaluating a combined CNEP and IPT (henceforth CNEP-IPT) mentoring program.

## Methodology

In the absence of mentoring data in PM [12, 31–33], this systematic scoping review will scrutinize data from specialities associated with Internal Medicine (IM) and Family Medicine (FM) or primary care [34–36] settings given evidence that mentoring data from FM and IM may be effectively extrapolated to the PM setting [6, 37–42].

To enhance the transparency and reproducibility, Krishna’s Systematic Evidence Based Approach [43–50] (SEBA) is adopted to guide this systematic scoping review. Systematic scoping reviews in SEBA utilise a constructivist perspective to map the complex topics of CNEP and IPT from multiple angles and acknowledge mentoring as a sociocultural construct built from the individual views and experiences of mentees, mentors and the host organization (henceforth stakeholders) [51]. A relativist lens allows for the historical, socio-cultural, ideological, and contextual factors impacting individual views and experiences of stakeholders to be considered within this review [52–56].

SEBA’s use of a systematized approach, supported by medical librarians from the Yong Loo Lin School of Medicine (YLLSoM) at the National University of Singapore and the National Cancer Centre Singapore (NCCS) and local educational experts and clinicians at the NCCS, the Palliative Care Institute Liverpool, YLLSoM and Duke-NUS Medical School (henceforth the expert team), allows for an accountable and reproducible approach to the search and review of data.

**Fig. 1:**
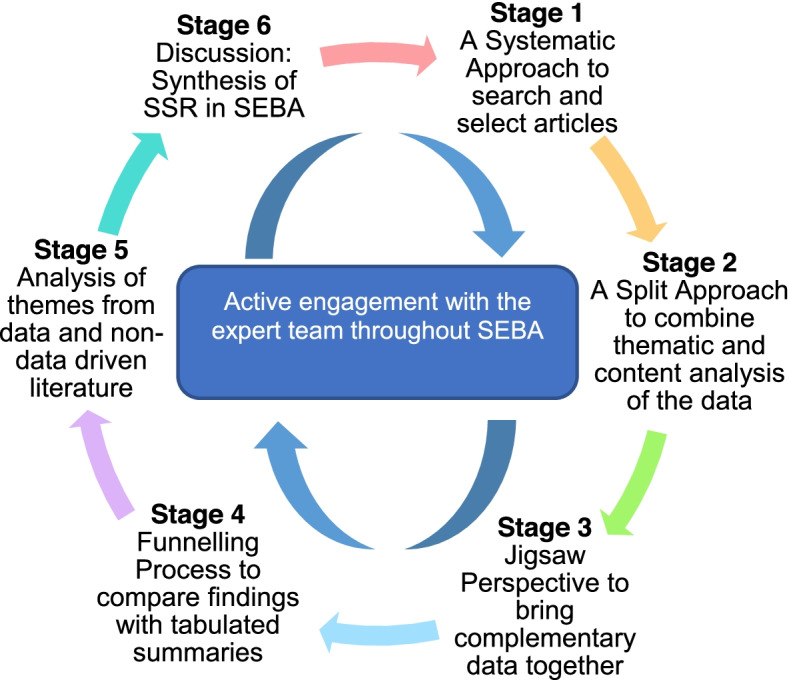
SEBA Process

SEBA’s use of the principles of interpretivist analysis [52, 54–56] enhance reflexivity and discussions in the Systematic and Split Approaches, the Jigsaw Perspective, the Funnelling Process, Analysis of themes from data and non-data driven literature, and the Synthesis of the systematic scoping review that make up SEBA’s six stages [43–50] outlined in Fig. 1*.*

The expert team was consulted at each stage of the SEBA process.

### Stage 1 of SEBA: systematic approach

i. *Determining title and background of review*

The expert team, stakeholders and the research team collaborated to determine the overall goals of the systematic scoping review and the population, context and concept to be evaluated.

This systematic scoping review in SEBA confines its review of CNEP and IPT amongst physicians and nurses, medical social workers, physiotherapists and/or occupational therapists [7].

ii. *Identifying the research question*

Guided by the population, concept and context (PCC), the teams also determined the primary research question to be “*what is known about CNEP and IPT?*” The secondary research questions were “*what are the features of CNEP and IPT?*” and “*is CNEP-IPT suitable for the PM setting?”*

iii. *Inclusion criteria*

All grey literature, peer reviewed articles, narrative reviews, systematic, scoping and systematic scoping reviews published between 1st January 2000 to 28th February 2021 were included in the PCC and a PICOS format was adopted to guide the research processes [57, 58]. See Table 1.

**Table 1:** PICOS, inclusion and exclusion criteria

PICOS	Inclusion Criteria	Exclusion Criteria
Population	*CNEP* • Undergraduate or post-graduate medical students or trainees in all years, residents, clinical fellows and/or attending physicians in clinical, medical, academia and/or research settings (for CNEP mentoring) • Internal Medicine Specialties (for articles based in clinical settings discussing CNEP) *IPT* • Undergraduate or post-graduate nursing, medical social work, physiotherapy and occupational therapy students or trainees in all years, nurses, medical social workers, physiotherapists and/or occupational therapists in clinical, medical, academia and/or research settings (only for articles discussing inter-professional mentoring) • Internal Medicine Specialties (for articles based in clinical settings) and Family Medicine (only for articles based in clinical settings discussing inter-professional mentoring)	• Non-healthcare related professions (e.g. Science, Veterinary), Psychology, Alternative and Traditional Medicine (including Chiropractic, Traditional Chinese Medicine) • Specialties other than Internal Medicine Specialties (for articles based in clinical settings discussing CNEP) • Specialties other than Internal Medicine Specialties (for articles based in clinical settings) and Family Medicine (only for articles based in clinical settings discussing inter-professional mentoring)
Intervention	• Electronic communication used to facilitate mentoring by senior, near-peer and peer mentors and its influence on the implementation and evaluation of mentoring program • Electronic platforms facilitating mentoring programs • Near-peer and peer- mentoring practices that support novice mentoring in its implementation and evaluation of mentoring programs • Interprofessional mentoring involving mentors and mentees who are medical, nursing, medical social work, physiotherapy and occupational therapy healthcare professionals and students	• Technology used but not in the medical mentoring communication process (for instance, ultrasound near-peer mentoring) • Mentoring for leadership as well as patient and family mentoring • Supervising, coaching, role-modelling, advising, tutoring, networking, sponsorship, wet-bench learning, tele-learning and skills-based learning • Poor characterisation of the way mentoring is conducted and how the mentees and mentors were involved
Comparison	• Comparison accounts of interprofessional, transprofessional, multiprofessional, interdisciplinary, transdisciplinary, multidisciplinary mentoring • Comparisons of the various definitions, descriptions, characteristics and roles of near-peer, peer- and e-mentoring in novice mentoring and their impact upon the mentoring process, mentoring relationship, mentor, mentee, host organization, and mentoring environment	
Outcome	• Definition and Characteristics of IPT or CNEP mentoring • Motivations, benefits and outcomes of IPT or CNEP mentoring, and their impact on the mentoring process, relationship, mentor, mentee, host organization and mentoring environment • Approach to nurturing IPT or CNEP mentoring • Methods and criteria of evaluation and assessment of IPT or CNEP mentoring • Challenges, limitations and knowledge gaps in IPT or CNEP mentoring	
Study design	• All study designs were included, including: o Mixed methods research, meta-analyses, systematic reviews, randomized controlled trials, cohort studies, case-control studies, cross-sectional studies, and descriptive papers o Grey Literature / electronic and print information not controlled by commercial publishing o Case reports and series, ideas, editorials, and perspectives • Articles in English or translated to English • Year of Publication: 1 January 2000 –28th February 2021

iv. *Searching*

To broaden the search, ensure a structured approach and reduce omission of critical papers, three separate search strategies were formulated to look for articles about CNEP, IPT and e-mentoring respectively. These search terms were developed based on the following definitions. Near-peer and peer mentoring is defined as “*informal dynamic advisory relationships within a group of individuals who are similar in experience, education level, and seniority*” [59]. E-mentoring is defined as an integration of “*synchronous (live video or instant messaging)* (60) *and asynchronous (email, online discussion board or social media)”* communication [30]. IPT refers to “*senior, near-peer and/or peer mentors who are medical, nursing, medical social work, physiotherapy and occupational therapy healthcare professionals or students supporting junior healthcare professionals and students in advancing their professional, clinical, personal and academic development*” [60].

Searches on seven bibliographic databases (PubMed, Embase, PsycINFO, ERIC, Cochrane Database of Systematic Reviews, Google Scholar and Scopus) and five grey literature databases (GreyLit, OpenGrey, Web of Science, Mednar and OpenDissertations) were carried out and included articles from 1st January 2000 to 28th February 2021. Articles published before year 2000 were not included given evidence that they often failed to clearly delineate distinct mentoring approaches such as leadership, patient, family, adolescent, group, peer, near-peer, novice and e-mentoring [61, 62], and conflate “mentoring” and practices such as teaching, tutoring, coaching, role modelling and supervision.

A manual search of related areas of interest, and an expanded search of the references of the included articles were also carried out. This revealed six additional articles of interest.

The three separate PubMed Search Strategies may be found in Additonal file 1: Appendix A.

v. *Extracting and charting*

Using the abstract screening tool, members of the research team independently reviewed the titles and abstracts found from each database to finalise the list of titles and summaries to be reviewed. Sambunjak, Straus and Marusic’s [63] approach to ‘negotiated consensual validation’ was used to achieve consensus.

### Stage 2 of SEBA: Split Approach

To enhance validity of the analysis, the Split Approach [64] was employed. The Split Approach [64] consists of concurrent thematic and directed content analysis of all the included articles by three independent teams. The first team summarised and tabulated the included full-text articles in keeping with recommendations drawn from Wong et al.’s [51] RAMESES publication standards: meta-narrative reviews and Popay et al.’s [53] “Guidance on the conduct of narrative synthesis in systematic reviews”. The tabulated summaries served to ensure that key aspects of included articles were not lost.

Concurrently, three members of the second team independently analysed the included articles using Braun and Clarke’s [65] approach to thematic analysis [64]. In phase 1 of Braun and Clarke’s [65] approach, the research team carried out independent reviews, ‘actively’ reading the included articles to find meaning and patterns in the data [66–70]. In phase 2, ‘codes’ were constructed from the ‘surface’ meaning and collated into a code book to analyse the rest of the articles using an iterative step-by-step process. As new codes emerged, these were associated with previous codes and concepts. In phase 3, the categories were organised into themes that best depict the data. An inductive approach allowed themes to be “*defined from the raw data without any predetermined classification*” [69]. In phase 4, the themes were refined to best represent the whole data set and were discussed. In phase 5, the research team discussed the results of their independent analysis online and at reviewer meetings. “*Negotiated consensual validation*” was used to determine a final list of themes [63].

A third team of three researchers employed Hsieh and Shannon’s [71] approach to directed content analysis to independently analyse the included articles. Analysis using the directed content analysis approach involved “*identifying and operationalizing a priori coding categories*” [71–76]. The first stage saw the research team draw categories from Krishna et al.’s [45] study titled “*Enhancing Mentoring in Palliative Care: An Evidence Based Mentoring Framework*”, to guide the coding of the articles in the second stage. Any data not captured by these codes were assigned a new code [72]. In keeping with *deductive category application*, coding categories were reviewed and revised as required [76].

### Stage 3 of SEBA: Jigsaw Perspective

The Jigsaw Perspective brings together the themes and categories identified in the Split Approach to provide a more holistic perspective of the available data. This process is overseen by the expert team and guided by six principles [77–79]:

1. Principle of pragmatism: in ensuring that the focus of the review remains upon the research question,
2. Principle of pluralism: in ensuring that all themes are included in the review,
3. Principle of historicity: in ensuring that the process is reproducible by including the review descriptions of the unfolding narrative,
4. Principle of contestation: in ensuring that all ‘conflicting data’ is considered,
5. Principle of reflexivity: in ensuring that throughout the review, reviewers continually reflect individually and as a team on the emerging findings, and
6. Principle of peer review: in ensuring that emerging findings are peer reviewed through use of the split review, peer reviewed data, and that that the stakeholders agree with the data and interpretation.

The process of creating themes/categories is derived from Phases 4 to 6 of France et al.’s [80, 81]‘s adaptation of Noblit et al.’s [82] seven phases of meta-ethnography. The themes and categories are contextualised by reviewing them against the primary codes and subcategories and/or subthemes they were drawn from [80, 81]. *Reciprocal translation* determines if the themes and categories can be used interchangeably.

*1) Themes identified through Braun and Clarke’s approach to thematic analysis*:

- Characteristics of CNEP and IPT
- Mentoring Stakeholders
- Stages of CNEP and IPT
- Assessment methods and criteria

*2) Categories identified through Hsieh and Shannon’s approach to directed content analysis*:

- Mentoring Nature
- Mentoring Stakeholders
- Mentoring Relationships
- Mentoring Approaches
- Mentoring Assessments

Here the combination of the themes/categories provides triangulation [83], improves audits and enhances the authenticity of the research [84]. The themes/categories were

- Characteristics
- Mentoring stages
- Roles of the host organization
- Assessments

### Stage 4 of SEBA: Funnelling

The themes/categories identified through the Jigsaw Process were reviewed and compared with the tabulated summaries in Additonal file 2: Appendix B to ensure no crucial information was left out.

Adapting Phase 5 of France et al’s [80, 81] approach, we adopted reciprocal translation to juxtapose the themes/categories identified in the Jigsaw Approach with the key messages identified in the tabulated summaries. This juxtaposition of themes/categories is important given that inclusion of grey literature, non-primary data driven articles, opinion pieces, editorials, essays, commentaries, letters, posters, oral presentations, forum discussions, interviews, blogs and surveys (henceforth non-evidence-based data) may sometimes over-generalise issues, conflate practices and fail to account for practical, clinical and contextual considerations. The verified themes/categories from the Funnelling Process then form the ‘line of argument’ process in the synthesis of the discussion portion in Stage 6 of the SSR in SEBA.

## Results

**Fig. 2:**
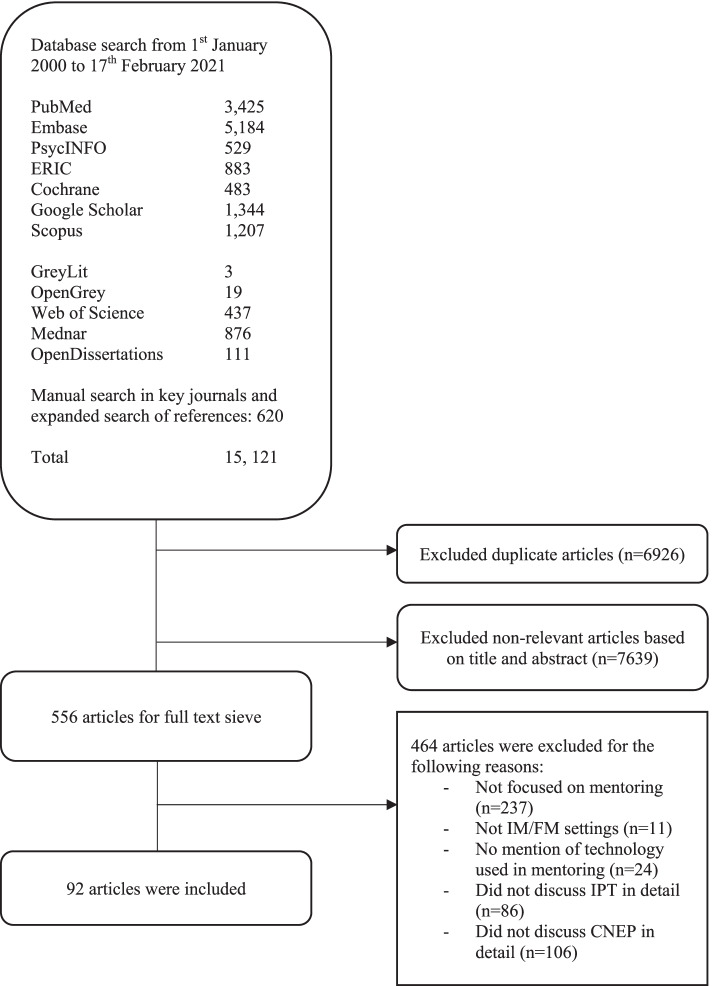
PRISMA Flowchart

A total of 15,121 abstracts were reviewed, 557 full text articles were evaluated, and 92 articles were included. See Fig 2.

A summary of the background, theoretical approach and methods, population characteristics, main empirical findings and insights drawn are highlighted in Additonal file 2: Appendix B*.*

### Theme/category 1: characteristics of CNEP and IPT

#### Similarities

CNEP and IPT provide timely, well-resourced, and high-quality [85–87] research [88–90], academic [91–94] and pastoral support [95–97] facilitated by a formal mentoring structure overseen by the host organization [98–100]. The motivation for most host organizations in supporting CNEP and IPT is to improve patient care and safety [98–100] by structuring mentoring programs and establishing guidelines, codes of conduct and standards of practice [101–106]. A consistent set of guidelines serve to confine mentoring practices within acceptable standards as mentoring programs try to accommodate to the individual goals [107–109], abilities [109, 110] and needs of mentors’ and mentees’ [13, 108, 111, 112], and to nurture a personalized, mentee-centric, non-judgmental, confidential and trusting environment [113–115].

The data garnered suggest that the similarities between CNEP and IPT are consistent with the critical aspects of novice mentoring and have likely evolved from novice mentoring roots, serving to emphasize the likelihood that they may be used to support novice mentoring relationships in the COVID-19 era and beyond when access to senior physician mentors is expected to remain limited.

#### Unique characteristics of CNEP

CNEP pivots on in-person face-to-face mentoring [103, 116–119] complemented by accessible electronic communication platforms [95, 120, 121] that facilitate synchronous [107, 122, 123] and asynchronous [30, 95, 124] communication. Use of accessible electronic communication platforms [95, 120, 121] allows for the rapid exchange of information [104, 116, 118] which circumvents geographical [107, 120, 125], logistical [107, 120, 124] and scheduling [103, 115, 120] restrictions and allows communication among mentees, near-peers and mentors to adapt according to circumstances and needs [126, 127]. Concurrently e-mentoring provides timely, flexible, and adaptive mentoring support [59, 105, 106, 118, 128].

Near-peer mentors provide mentees with an alternative source of professional, personal, research and clinical support [113–115, 128], while they in turn gain learning opportunities [129], confidence [130], communication skills and a chance to ‘pay it forward’ [111, 122, 131]. CNEP also helps attenuate the sense of hierarchy within the program [59, 105, 118].

#### Unique characteristics of IPT

IPT is reliant on each team member possessing effective interprofessional communication and teamwork skills [24, 90, 130] within an ‘open’ environment, in contrast to traditional hierarchies amongst the various professions [89, 132, 133].

From these findings, it is also evident that while CNEP and IPT possess unique characteristics, these elements are able to build upon each other to enhance the effectiveness of a mentoring program and a holistic mentoring environment.

### Theme/category 2: stages of CNEP and IPT

CNEP and IPT both exhibit the presence of mentoring stages first described in novice mentoring, reaffirming the notion that they could be used to support novice mentoring programs. These stages include the pre-mentoring stage, mentoring process and the post-mentoring stage.

#### Pre-mentoring stage

The pre-mentoring stage includes recruitment of mentors and mentees [107, 112, 134], evaluation of the needs, skills and knowledge of participating mentors and mentees [100, 120, 135], determining appropriate instructional approach and content [7, 131, 136], skills training [122, 131] and communications and assessment platforms [98, 101, 106, 114], and agreeing upon the codes of conduct and standards of practice [130, 134, 137]. These elements are overseen by the host organization [20].

The host organization also determines the matching process [7, 134, 138]. To match mentees with mentors, host organizations often employ ‘criterion based matching’ in CNEP and IPT [24]. ‘Criterion based matching’ determines the complementariness of the mentors’ and mentees’ goals, motivations, specific needs, working styles, interests, hobbies, work-life balance priorities [92, 102, 139] and personality traits [102, 112, 139]. Within CNEP, mentees are often matched to near-peers and mentors of the same specialty [107, 112, 122] to support their academic needs [134].

However, given the venture towards inter-professional mentoring, important considerations in the matching process to best suit the dynamic, complex and multi-level mentoring relationships within CNEP-IPT still remain unclear.

#### Mentoring process

The mentoring process begins once a mentee and mentor agree to a match and commence on a mentoring relationship with each other [140–143]. At this stage mentees and mentors seek to build rapport [134] and agree upon specific mentoring goals, expectations, codes of conduct [20, 92, 127], roles and responsibilities [96, 101, 109, 135] and timelines [92, 134, 141]. These meetings are also complemented by synchronous and asynchronous verbal and/or written communication [30, 127].

CNEP programs may employ video conferencing in the initial face-to-face meeting [20, 127, 144].

#### Post-mentoring stage

The post-mentoring stage involves assessments of the mentoring process [20, 123, 124], the mentoring relationship [100, 123, 145], whether the host organisation has fulfilled its roles and responsibilities [110], and if the mentoring goals were achieved [88, 124, 146]. Such evaluations help direct future improvements to mentoring programs [97, 147, 148] and may occur longitudinally [112, 146, 149].

### Theme/category 3: roles of the host organization

The host organizations of CNEP and IPT programs play crucial roles in overseeing and running the mentoring programs. The roles are described in Table 2 for ease of reference*.*

**Table 2:** Roles of the Host Organization

Role of Host Organisation		References
**1**	*Design and Coordination*	
1.1	Conduct large-scale context-specific research into the design of mentorship programs.	[7, 26–29, 148, 150, 151]
1.2	Consider collaborative ventures with other organizations to pool resources in the implementation of a large-scale and high-impact mentoring program. This may increase the applicability of the program to a variety of settings as context-specific considerations are made during the designing process.	[7, 24, 26–29, 151]
1.3	Recruitment of suitable and willing mentors, near-peers and mentees and establishing mentor and mentee registries.	[7, 26–29, 107, 110, 112, 122, 125, 130, 134, 145, 151, 152]
1.4	Establish the overall mentoring structure, process, content, guidelines, codes of conduct and standards of practice to prevent ethical, legal and professional lapses and misconduct among mentors, near-peers and mentees and reduce the risk of mentoring abuse.	[7, 24, 26–30, 59, 98, 101–106, 108, 109, 111–114, 120, 122, 124, 125, 127–131, 134–137, 145, 146, 149, 151, 153–160]
1.5	Facilitate matching of mentors, near-peers and mentees.	[7, 20, 24, 26–29, 92, 102, 107, 110, 112, 122, 125, 134, 139, 144, 148, 150–152, 159]
1.6	Provide periodic reminders to mentors, near-peers and mentees to encourage regular meetings.	[134, 141, 144, 152].
1.7	Sustain mentoring programs by providing financial, administrative, logistical support, thereby sustaining a suitable mentoring environment. These include: - protected mentoring time [7, 26–30, 59, 89, 111, 122, 124, 125, 127, 131, 136, 148, 150, 151, 153–155] for minimum meeting frequencies to be achieved comfortably [30, 100, 108, 109, 113, 119, 122, 123, 125, 130, 134, 145, 146, 149, 153, 154] - formal recognition for mentors’ and mentees’ efforts [7, 24, 26–29, 151, 159] through promotions, awards, and reduced workloads [7, 24, 26–28, 151, 159]. - independent, fair and transparent recognition for near-peers [110, 111, 122, 131] - coordinate the various stages of mentoring [7, 24, 26–28, 151, 159] - create a safe environment for mentees to voice their concerns and feedback, forward ideas and share experiences [7, 24, 26–28, 124, 134, 151, 159] - providing suitable mentoring environments for interprofessional collaboration on research and academic projects [26, 27, 151, 159] - introducing the use of electronic platforms for mentoring such as e-mail [85, 152, 158], social networking [14, 103, 104, 111, 116, 119, 122, 130, 136, 145, 157], instant messaging, tele-conferencing, discussion forums and micro-blogging [14, 85, 99, 100, 104, 105, 111, 116, 119, 122, 123, 128, 129, 134, 135, 146, 153, 155, 157, 161–163], and to put in place proper security measures such as end-to-end encryptions to these platforms and resources [99, 108, 119, 152, 153]	[7, 24, 26–30, 59, 111, 122, 124, 125, 127, 131, 134, 136, 151, 153–155, 159]
**2**	*Conducting Training*	
2.1	Evaluation of the need, skills and knowledge of mentors, near-peers and mentees.	[100, 120, 122, 127, 130, 135, 146]
2.2	Organize training programs for mentors, near-peers and mentees including - leadership skills [122, 131] - communication and collaborative skills [7, 111, 122, 127, 129, 131, 136] - team management skills [111, 129, 131] - navigating challenging situations [154] - providing timely, effective and holistic support [7] - nurturing effective mentor-mentee relationships [154] - assessing mentees [7, 108, 120, 122, 134] - providing feedback [7, 108, 120, 122, 134] - establishing codes of conduct and standards of practice - promoting interprofessional teamwork [7, 24, 26–29, 89, 148, 150, 151, 159] - teaching electronic etiquette [99, 108, 119, 152, 153] cyberspace security and online professionalism [14, 98, 99, 101, 104, 105, 108, 114, 120, 125, 128].	[7, 27–29, 108, 111, 120, 122, 127, 129, 131, 134–136, 140, 145, 148, 150, 154]
**3**	*Evaluation*	
3.1	Evaluate mentors’, near-peers’ and mentees’ constantly evolving needs, goals and abilities [30, 100, 108, 109, 113, 122, 125, 130, 146, 153], mentoring effectiveness and efficiency, relationships, approaches and environment [7, 24, 26–30, 108, 122, 131, 136, 151, 159].	[7, 24, 26–30, 100, 108, 109, 113, 122, 125, 130, 131, 136, 146, 151, 153, 159]
3.2	Conduct post-mentoring evaluation.	[20, 24, 26–30, 85, 88, 95–100, 107, 108, 110–112, 115, 122–131, 134–136, 144–146, 149, 151–153, 156–159, 163, 164]

Evidently, the role of the host organization is integral in ensuring effective support of complex mentoring relationships within CNEP-IPT and in circumnavigating ethical concerns regarding the misappropriation of mentee’s work, disregard for the needs of mentees, and even bullying [15].

### Theme/category 4: assessment methods and criteria

Assessment of CNEP and IPT mentoring programs revolve around the mentee’s perspectives and experiences [107, 111, 125], but have increasingly adopted a more holistic perspective by including mentors [122, 131, 157] and host organizations [110] in assessments. These assessments often take the form of objective or subjective self-assessments using pre- and/or post- questionnaires and surveys [97, 147, 165], interviews [87, 93, 142], formative and summative examinations [127, 153], work-based assessments [86, 87], portfolio assessments [107, 108] and/or journaling [97, 138]. Most of these tools have not been validated [163, 166, 167].

#### Assessment criteria

The success of IPT mentoring programs is evaluated based on impact on mentor and mentee welfare, effectiveness and efficiency of the program, project outcomes, research output and improvements in patient care [26, 151, 159]. The evaluation criteria for CNEP mentoring programs are summarised in Table 3.

**Table 3:** A summary of prevailing evaluation criteria for CNEP mentoring

	Evaluation Criteria	References
**1**	*Pre-mentoring Stage*	
1.1	Mentor, near-peer and mentee training	[110]
1.2	Establishment of overall mentoring structure, process, guidelines, codes of conduct and standards of practice	[110, 127]
1.3	Formal matching process	[110]
**2**	*Mentoring Process*	
2.1	Communication	
2.1.1	Frequency of communication	[20, 111, 125, 144, 157]
2.1.2	Usability and accessibility of in-person and online communication platforms	[30, 124, 153, 156, 157]
2.2	Mentees’ and mentors’ adherence to guidelines and codes of conduct	[20, 111, 123, 124, 127, 144, 153]
2.3	Mentees’ and mentors’ active participation in mentoring activities	[20, 111, 123, 124, 127, 144, 153]
**3**	*Post-mentoring Stage*	
3.1	Mentees’ and mentors’ quality of performance, assignments and projects	[20, 111, 123, 124, 127, 144, 153]
3.2	Improvements in patient care and safety	[98–100, 110, 111, 127, 131, 145, 156]
**4**	*Criteria relevant to more than one stage*	
4.1	Host Organisation	
4.1.1	Oversight over mentoring programs	[110]
4.1.2	Provision of financial, administrative, logistical, technical and medico-legal support	[110]
4.2	Mentors/Near-Peer Mentors	
4.2.1	Experiences as senior or near-peer mentors	[20, 30, 111, 124, 135, 144–146, 153, 156, 157]
4.3	Mentoring Relationships	
4.3.1	Open, trusted and authentic relationships	[20, 85, 111, 144, 145]
4.3.2	Fulfilment of previously agreed goals, expectations, timelines, codes of conduct and roles and responsibilities within the mentoring relationship	[20, 111, 144]
4.3.3	Overall satisfaction of mentors, near-peers and mentees with mentoring relationships	[20, 100, 111, 144, 146]
4.3.4	Mutual appreciation	[20, 111, 144]
4.4	Growth of Mentors, Near-Peers and Mentees	
4.4.1	Personal growth	[20, 30, 85, 95–97, 100, 107, 108, 110, 111, 115, 122, 126, 130, 131, 136, 144, 149, 158, 164]
4.4.2	Professional and career development	[30, 96, 99, 100, 110–112, 122, 127, 129, 131, 136, 144, 152, 158, 164]
4.5	Evaluating the Assessments Used	
4.5.1	Evaluation of the inherent biases, subjectivity and reliability of self-assessments	[124]
4.5.2	Evaluation of the validity of existing instruments used for assessment	[124, 153, 156]

Holistic assessment approaches are especially important in a CNEP-IPT program given the multi-level nature of mentoring and also the large number of stakeholders involved. Longitudinal assessments are also crucial in the continual improvement and development of this novel mentoring approach.

### Stage 5 of SEBA: analysis of evidence-based and non-data driven literature

To evaluate the impact of grey literature and opinion, perspectives, editorial, letters and non-data based articles (henceforth non-data driven group) drawn from bibliographic databases upon the systematic scoping review, evidence-based data from bibliographic databases (henceforth evidence-based publications) were separated from the non-data driven group and both groups were thematically analysed separately. The themes from both groups were compared and found to be similar, suggesting that the non-data driven publications are unlikely to steer the systematic scoping review away from evidenced data.

### Stage 6 of SEBA: Synthesis of the systematic scoping review

The systematic scoping review produced from consolidating the themes, categories and tabulated summaries was guided by the Best Evidence Medical Education (BEME) Collaboration guide [168] and the STORIES (Structured approach to the Reporting In healthcare education of Evidence Synthesis) statement [169].

## Discussion

Recent research provides promising accounts of programs adopting a similar CNEP-IPT concept, which employ “systems of mentors” comprising “senior colleagues, teachers, peers, as well as junior colleagues and students” [7, 29, 60, 89], demonstrating a high likelihood of program success, provided deeper research and understanding on the topic can be derived. This systematic review in SEBA provides a structured approach to deliberating important considerations in the designing and evaluation of a CNEP-IPT program.

In answering its primary and secondary research questions, this review suggests that CNEP and IPT mentoring programs share similarities in their practice, structure, stages, mentoring goals, codes of conduct, assessment processes and mentoring environments that ought to allow a melding of these approaches and the creation of a combined CNEP-IPT mentoring program [24, 142, 143, 159]. This combined approach appears equipped to provide timely, personalized, accessible, and holistic support to mentees while ensuring effective policing of compliance to established Codes of Practice and agreed goals, expectations, timelines, and roles and responsibilities of stakeholders. The data here also suggests that a combined CNEP-IPT mentoring program would facilitate effective adaptations to mentoring support amid changing stakeholder related circumstances [13, 108, 111, 112], goals [107–109] and availabilities [109, 110].

It is also clear from our findings that a combined CNEP-IPT mentoring program would need to be part of a formal mentoring program, designed, supported, and overseen by the host organization [98–100]. This would ensure that mentoring guidelines, roles and responsibilities, and codes of practice are agreed upon [101–106], and also that matching processes [7, 134, 138], communication platforms [98, 101, 106, 114] and assessment programs are effectively coordinated [98, 101, 106, 114] to maintain patient care and safety [98–100]. This is even more crucial given the unconventional and novel roles of near-peer mentors in providing alternative support for mentees outside of their specific disciplines, which are not as well understood and may be prone to ethical lapses. A formal well-structured program also delineates, guides, and supports progress through the mentoring stages and in the nurturing of an effective mentoring environment [89, 132, 133]. This would then facilitate mentor and mentee training [122, 131] particularly when healthcare professionals from different specialist backgrounds are enlisted to coordinate provision of timely, personalised and appropriate mentoring support while maintaining a consistent mentoring approach [24, 90, 130]. The need for a structured approach in mentor training is especially prevalent as many of the assessment tools remain unvalidated, non-standardised and not holistic and thus reliant upon the knowledge, skills and attitude of the mentors. Concurrently, mentor training would also enhance teamworking within the IPT portion of the mentoring team and amongst near pear mentors to help attenuate the hierarchy within the program [59, 105, 118] and foster more open mentoring relationships [86, 97].

The notion of a synergistic relationship between these mentoring approaches also requires further study given implications upon mentoring dynamics in the presence of multiple stakeholders. Mentoring relationships in CNEP-IPT may be more challenging compared to traditional ones given inherent hierarchical boundaries that exist both in terms of disparities in seniority and qualifications, and also across healthcare disciplines. Yet it is also worth noting that with time and the promotion of inter-professional collaboration and teamwork among stakeholders, CNEP-IPT may help to break down these historical barriers. This gives rise to considerations on how mentors, near peers and mentees can be best matched for the task. As a result, this review suggests that a combined CNEP-IPT mentoring program ought to be designed, supported, overseen and assessed by the host organization and be part of a formal program. Indeed the host organization plays a key role in the effective running of the proposed CNEP-IPT mentoring program with the roles and responsibilities of the host organization set out in Table 2. Should the aforementioned be achieved, a combined CNEP-IPT mentoring program could serve as a platform to nurture interprofessional ties crucial to team-based care in Palliative Medicine.

## Limitations

Despite efforts to enhance the reproducibility and transparency of the systematic scoping review, gaps in the methodology and analysis persist. While we have conducted three separate search strategies using a two-tiered approach of both independent searching of selected databases by our expert team and an expanded search of reference lists of publications and manual searches, important papers may still have been missed. Similarly, while use of the ‘Split Approach’ and tabulated summaries in SEBA allowed for triangulation and ensured a holistic perspective was constructed from different and diverse perspectives, inherent biases amongst the reviewers would still impact the analysis of the data and construction of themes. Moreover, SEBA is not evidenced as yet and is time-consuming, raising questions as to its viability and concerns of the need for careful balancing between the promised benefits and the sustainability of continued involvement of the expert teams who were involved in all stages of SEBA.

The use of thematic analysis to review the impact of non-evidence-based data improves transparency in the synthesis of the discussion, however the inclusion of these data may still bias results and provide opinion-based views with a ‘veneer of respectability’ despite a lack of evidence to support them. This raises the question as to whether non-evidence-based data should be accorded the same weight as published literature.

## Conclusion

In addressing its research questions, this systematic scoping review in SEBA offers a glimpse into the future of mentoring in PM but also raises a word of caution. While synergy between CNEP and IPT built on a common ancestry rooted in novice mentoring is evident, there are many aspects of the process that require further study. However, evidence of the desire to continue promoting mentoring in these difficult circumstances underscores its reputation and role within PM. We look forward to engaging in this developing field as advances in the understanding of dynamics, support and oversight within these relationships begin to take shape and help mould a structured approach to this form of mentoring within PM and beyond.

## Supplementary Information

**Additional file 1.** Search Strategies for PubMed.

**Additional file 2.** Summary of Included Articles.

## Data Availability

All data generated or analysed during this study are included in this published article.

## Abbreviations

PM: Palliative Medicine
CNEP: Combination of a Novice Mentoring, Near Peer, Peer and E-mentoring approach
IPT: Interprofessional Team
IPT: Interprofessional Mentoring
SSR: Systematic Scoping Review
IM: Internal Medicine
FM: Family Medicine
SEBA: Systematic Evidence Based Approach
YLLSoM: Yong Loo Lin School of Medicine
NCCS: National Cancer Centre Singapore
PCC: Population, Concept, Context
PICOS: Population, Intervention, Comparison, Outcome, Study Design
