# Supplementary material for: Mentoring in palliative medicine in the time of covid-19: a systematic scoping review: Mentoring programs during COVID-19
Source: BMC Med Educ. 2022 May 11;22:359. doi: 10.1186/s12909-022-03409-4 (PMC9094135; doi:10.1186/s12909-022-03409-4)
Supplement: Supplementary file 1 — Additional file 1. Search Strategies for PubMed. [file 12909_2022_3409_MOESM1_ESM.pdf]

Appendix A: Search Strategies for PubMed

**Topic of interest**	**PubMed Search Strategy**
IPM	(“Interprofessional Relations”[MeSH] OR “Interdisciplinary Communication”[MeSH] OR “Interdisciplinary Studies”[MeSH] OR “interprofessional”[tiab] OR “inter-professional”[tiab] OR “transprofessional”[tiab] OR “trans-professional”[tiab] OR “multiprofessional”[tiab] OR “multi-professional”[tiab] OR “interdisciplinary”[tiab] OR “inter-disciplinary”[tiab] OR “transdisciplinary”[tiab] OR “trans-disciplinary”[tiab] OR “multidisciplinary”[tiab] OR “multi-disciplinary”[tiab])<br><br>AND<br><br>(“mentoring”[MeSH] OR mentors[MeSH] OR mentor[tiab] OR mentors[tiab] OR mentoring[tiab] OR mentorship[tiab] OR “mentoring structure”[tiab] OR “mentoring structures”[tiab] OR “mentoring relationship”[tiab] OR “mentoring relationships”[tiab] OR “mentoring environment”[tiab] OR “mentoring environments”[tiab])<br><br>AND<br><br>(“Physicians”[MeSH] OR “Schools, Medical”[MeSH] OR “Medicine”[MeSH] OR “Students, Medical”[MeSH] OR “medical student”[tiab] OR “medical students”[tiab] OR “medical school”[tiab] OR “medical schools”[tiab] OR “medicine”[tiab] OR “doctor”[tiab] OR “doctors”[tiab] OR “physician”[tiab] OR “physicians”[tiab]<br><br>OR “Nurses”[MeSH] OR “Nursing”[MeSH] OR “Schools, Nursing”[MeSH] OR “Students, Nursing”[MeSH] OR “nurse”[tiab] OR “nurses”[tiab] OR “nursing”[tiab] OR “nursing student”[tiab] OR “nursing students”[tiab] OR “nursing school”[tiab] OR “nursing schools”[tiab]<br><br>OR “Social Work Department, Hospital”[MeSH] OR “medical social work”[tiab] OR “medical social worker”[tiab] OR “medical social workers”[tiab] OR ((“Social Work”[MeSH] OR “social work”[tiab] OR “social worker”[tiab] OR “social workers”[tiab]) AND (“Medicine”[MeSH] OR “Hospitals”[MeSH] OR “medical”[tiab] OR “medicine”[tiab] OR “hospital”[tiab]))<br><br>OR “Physical Therapists”[MeSH] OR “Occupational Therapists”[MeSH] OR “Occupational Therapy”[MeSH] OR “Physical Therapy Specialty”[MeSH] OR “physical therapist”[tiab] OR “physical therapists”[tiab] OR “physiotherapist”[tiab] OR “physiotherapists”[tiab] OR “occupational therapist”[tiab] OR “occupational therapists”[tiab] OR “physical therapy”[tiab] OR “physiotherapist”[tiab] OR “occupational therapy”[tiab])
CNEP mentoring	("Medicine"[Mesh] OR "Education, Medical, Undergraduate"[Mesh] OR "Schools, Medical"[Mesh] OR medicine[Title/Abstract] OR “medical

	education"[Title/Abstract] OR "medical school*"[Title/Abstract} OR "medical student"[Title/Abstract])<br><br>AND<br><br>(mentor*[Title/Abstract] OR "Mentors"[Mesh] OR "Mentoring"[Mesh] OR e-mentor*[tiab] or ementor*[tiab] OR cybermentor*[tiab] OR telementor*[tiab] OR tele-mentor*[tiab])<br><br>AND<br><br>("peer"[Title/Abstract] OR "near-peer"[Title/Abstract] OR group[Title/Abstract])<br><br>AND<br><br>(virtual[tiab] OR online[tiab] OR cyber[tiab] OR distance[tiab] OR "long-distance"[tiab] OR electronic[tiab] OR internet[tiab] OR web[tiab] OR blend[tiab] OR blended[tiab] OR skype[tiab] OR video[tiab] OR "Computer-assisted instruction"[MeSH] OR "Education, Distance"[MeSH] OR "Electronic mail"[MeSH] OR "Text Messaging"[MeSH] OR "social media"[MeSH] OR "Social Networking"[MeSH] or "Internet"[Mesh]))
E-mentoring	((("Computer-assisted instruction"[MeSH] OR "Education, Distance"[MeSH] OR "Electronic mail"[MeSH] OR "Text Messaging"[MeSH] OR "Mobile Applications"[Mesh] OR "Smartphone"[Mesh] OR "Videoconferencing"[Mesh] OR "social media"[MeSH] OR "Social Networking"[MeSH] OR "Internet"[Mesh] OR "Telephone"[Mesh] OR virtual[tiab] OR online[tiab] OR cyber[tiab] OR distance[tiab] OR remote[tiab] OR electronic[tiab] OR internet[tiab] OR web[tiab] OR skype[tiab] OR facebook[tiab] OR WhatsApp[tiab] OR video[tiab] OR videoconference[tiab] OR video conference[tiab] OR mobile[tiab] OR telephone[tiab] OR handphone[tiab] OR smartphone[tiab] OR social media[tiab] OR Tele-education[tiab] OR email*[tiab] OR e-mail*[tiab] OR electronic mail*[tiab]) AND ("Mentors"[Mesh] OR "Mentoring"[Mesh] OR Mentor[tiab] OR mentors[tiab] OR mentorship[tiab] OR mentorships[tiab] OR mentee[tiab] OR mentees[tiab] OR mentoring[tiab] OR mentor-mentee[tiab] OR mentee-mentor[tiab] OR mentored[tiab])) OR (e-mentor*[tiab] OR electronic mentor*[tiab] OR tele-mentor*[tiab] OR cyber-mentor*[tiab] OR remote mentor*[tiab] OR online mentor*[tiab]))<br><br>AND<br><br>("Education, Medical"[Mesh] OR "Faculty, Medical"[Mesh] OR medical[tiab] OR clinical[tiab] OR medicine[tiab] OR "Telemedicine"[Mesh] OR telemedicine[tiab] OR tele-medicine[tiab])
