# Supplementary material for: Mentoring in palliative medicine in the time of covid-19: a systematic scoping review: Mentoring programs during COVID-19
Source: BMC Med Educ. 2022 May 11;22:359. doi: 10.1186/s12909-022-03409-4 (PMC9094135; doi:10.1186/s12909-022-03409-4)
Supplement: Supplementary file 2 — Additional file 2. Summary of Included Articles. [file 12909_2022_3409_MOESM2_ESM.pdf]

Appendix B: Summary of Included Articles

Author	Year	Title	Background	Theoretical approach and methods	Population characteristics	Main empirical findings	Insights drawn
Ahmad S. Alamro & Susie Schofield	2012	Supporting traditional PBL with online discussion forums: A study from Qassim Medical School	The DF (also known as a discussion board, bulletin discussion or forum) 'allows learners and tutors to engage in an extended, structured dialogue on topics of relevance to their course of study'. Blending F2F with online DFs can address a variety of learning styles by offering instructional materials in different formats.	Type of study:<br>Pilot Interventional Study with Qualitative Feedback<br><br>Methodology:<br>The 5-week PBL course was facilitated by 14 tutors (10 males, 4 females), each assigned to one PBL group. During the block, five PBL scenarios were given. Each week, students attended two sessions for every scenario (problem), Saturday and Wednesday, respectively.<br>Membership of the 14 online groups matched the F2F groupings.	First Year Medical Students in a 5-week block	The majority (86% of those answering the question) agreed that using the DFs increases contact time between students, and 85% agreed that it helped them sharing knowledge. Students also found that the DFs enhanced the interaction with their tutors (60%). Most (89%) agreed that utilizing the DF gave them the opportunity to express their opinions. Most (93%) of the tutors considered the virtual PBL room as a continuity of the F2F PBL and felt it helped students find useful resources. Less than a fifth of the students were negative about the intervention (Table 3). Most of the tutors (93%) were satisfied from using the DFs, and 79% look forward to having the same intervention in the future blocks.	• Students and tutors perceived the online addition to the F2F PBL positively. Almost all the tutors agreed that using the DFs enhanced interactivity and student–student and student–tutor collaborations.<br>However, a third did not agree that they considered the virtual PBL room as a continuity of their F2F PBL.
Actrn	2019<br>`	From Medical Students to Junior Doctors - Feasibility of a Mentor supported transition through the "Resident Ready Network"	The near-peer mentoring program, the Resident Ready Network (RRN) has been developed in consultation with medical students, doctors and researchers in South-East Queensland. The RRN will provide students with a semi-structured support network to develop a professional relationship with a junior doctor. The RRN is embedded and supported by the Chronus digital mentor platform and linked in through University of Queensland (UQ) services and processes.<br>Chronus is an online mentoring platform, which provides a framework allowing users to create, implement and manage mentoring programs.	Type of study:<br>Interventional with Qualitative Feedback<br><br>Methodology:<br>Participating mentors and mentees will sign up to become a member on a digital mentoring platform called 'Chronus'. The platform will be used for participant contact and data collection points. The groups participating in the RRN will receive approximately 12 months of active near-peer mentoring.	Mentees : will be excluded if they are not a UQ fourth year medial student enrolled in the Metro South Division of UQ<br>Mentors : will be excluded if they are not a junior doctor at the PAH.	N.A.	N.A.

			The primary outcome is the feasibility of the RNN - to describe the RRN framework, matching, and experience with the RRN. Chronus resources, use of the the Chronus platform, and contacts made with the RRN team will also be collected to inform of overall feasibility and acceptability. Pair speicifc intricacies will be explored, for example length of mentor relationships, participant retention, number of meetings per year and the mode, and content and the style of mentoring.				
Akinla, O.	2018	A systematic review of the literature describing the outcomes of near-peer mentoring programs for first year medical students	Describe the outcomes of near-peer mentoring schemes for first-year medical students in the transition phase.	Type of study: Systematic Review<br><br>Methodology:<br>A search of different electronic databases was carried out, using the search terms peer, buddy, mentor*, counsel*, advise*, tutor*, student, medical, school. 1861 articles were identified, however only 5 studies met the inclusion criteria- primary mentees should be first-years, and mentors must be inclusive of second-years but not limited to them. In reporting this paper, the PRISMA guidelines were followed.	Mentees must be 1st year student, mentors must be near peers	Published material on near-peer mentoring for medical students is scarce. Three outcomes for peer mentoring were identified- professional and personal development, stress reduction, and ease of transitioning. Incidentally, peer-mentoring was also found to have facilitated the development of personal and professional attitudes in the mentors. The quality of the evaluation methods in the studies was however low to moderate	• Assessment methods included questionnaires and interviews<br>• Near-peer-mentoring is a way of promoting professional and personal development<br>• Near peer mentoring aids transition and maintains well-being of first-year medical students. However, larger, better quality longitudinal studies, are needed to ascertain its true value for these students.
Armstrong et al	2004	Innovative strategic Canadian research training from Tomorrow's Research Cardiovascular	Cardiovascular research training is experiential, and "skills" are traditionally acquired through a master–apprentice paradigm. The complexity of contemporary clinical research requires a new model for	Type of study: Descriptive paper<br><br>Methodology:<br>The main components of the program include	19 trainees from a broad cross-section of disciplines, integrating 2 University of	This report describes the vision, mission, core values, objectives, design and curriculum of the program. Our vision is the development of a new generation of cardio-vascular research clinician–scientists, with particular	• Through the medium of 1-hour weekly videoconferences, the curriculum cycles through case studies, seminars and a journal

		Health Care Professionals (TORCH)	research training. Facilitated through a Strategic Training Program Initiative, the Canadian Institutes of Health Research (CIHR), with its partners the Alberta Heritage Foundation for Medical Research and the Heart and Stroke Foundation, supported the Universities of Alberta and Calgary to create a new and innovative training model. Tomorrow's Research Cardiovascular Health Profession-als (TORCH) is an integrated 2-year program for health care professionals from diverse disciplines to be men-tored toward careers as leaders in translational cardio-vascular research, applying discovery to human health.	acquisition of a graduate degree or post-doctoral training, mentored research and a specialized core curriculum that unfolds over a 2-year period.	Alberta campuses to become new generation of cardiovascular research clinician-scientists	emphasis on thought, leadership and collaboration. The program incorporates 4 core values: innovation and discovery, a translational and transdisciplinary focus, an emphasis on collaboration and integration of research concepts, and the teaching of a core body of research knowledge coupled with real-world "survival" skills.	club in focused areas of cardiovascular research. Men-tors in the TORCH program have diverse backgrounds that epitomize the transdisciplinary translational aspects of the program and are chosen for their proven record of research accomplishment and prior history of successful mentoring.
Amy Prunuske, BreAnna Houss, & Anna Wirta Kosobuski	2019	Alignment of roles of near-peer mentors for medical students underrepresented in medicine with medical education competencies: a qualitative study	Medical student learning experiences should facilitate progressive development of competencies required for practice. Medical school training opportunities have traditionally focused on acquiring medical knowledge and patient care competencies while affording less opportunity to receive feedback on practice-based improvement and system-based practice competencies. The Prematriculation program at the University of Minnesota Medical School Duluth Campus (UM MSD) utilized near-peer mentors to support the transition of students underrepresented in medicine, including American Indian/ Alaska Natives (AI/AN) and those from rural backgrounds, into medical school. The purpose of this study is to better define the role of near-peer mentors and explore the alignment of near-peer mentorship with the ACGME core competencies.	Type of study: Qualitative study<br><br>Methodology: An important component of the Prematriculation program, designed to prepare incoming under- represented students for medical school, was the inclusion of near-peer mentors. The six near-peer mentors participated in semi-structured interviews or focus groups within 1 year of serving as a near-peer mentor. Themes emerged from open-coding of the transcripts.	Undergraduate students	The near-peer mentors drew on their own experiences to transmit information that supported the socialization of the matriculating students into medical school. Direct benefits to the mentors included solidifying their own understanding of medical knowledge and execution of procedural skills. Mentors provided examples of benefits related to their own development of interpersonal communication and professionalism skills. Operating in the context of the program provided opportunities to engage mentors in practice-based improvement and system- based practice.	• The Prematriculation program at the University of Minnesota Medical School Duluth Campus (UM MSD) utilized near-peer mentors to support the transition of students underrepresented in medicine, including American Indian/ Alaska Natives (AI/AN) and those from rural backgrounds, into medical school. The near-peer mentors drew on their own experiences to transmit information that supported the socialization of the matriculating students into medical school.<br>• Mentors see improvement in

							medical knowledge and procedural skills, and development of interpersonal communication and professionalism skills. Serving as near-peer mentor also offers significant benefits to medical students from backgrounds underrepresented in medicine in reducing anxieties and uncertainties whilst matriculating into the school.
Barton	2006	Clinical mentoring of nurse practitioners: the doctors' experience	The clinical development of nurse practitioners (NPs) has historically been dependent on mentorship from medical practitioners, yet their experience of this mentorship is generally unexplored. NPs have an ambiguous relationship with medicine as they have been dependent on medical mentorship to develop clinical skills, and they substitute into roles traditionally associated with medical practice. Consequently, NPs challenge professional boundaries and present particular concerns to their medical mentors. Practitioner ethnography examined the experiences of medical mentors, nurse practitioner students and academic staff during a clinical degree programme.	Type of study:<br>Qualitative study<br><br>Methodology:<br>A qualitative longitudinal participant observation of a group of participants in a part-time clinical degree programme (BSc (Hons) Nurse Practitioner) was conducted, considering both ethnography and prolonged comparative latent content analysis, characterized by an evolving systematic inductive data mapping and coding of 990 textual segments (identified from the 35 interview transcripts) and 55 detailed field notes episodes.	Medical mentors, nurse practitioner students and academic staff	This paper reports specifically on the medical mentors, focusing primarily on their professional authority relationship with their students and on their experience of imparting and sharing clinical knowledge. These experiences fell into three perspective stages, the provisional perspective, transitional perspective, and final perspective.	• Medical mentors were instrumental to the advanced clinical role of the student NP. This resulted in a conflicting experience of promoting a clinical role that challenged traditional medical authority. The effect of this was a cautious renegotiation of professional boundaries. In future NP students (and their academic teachers) need to acknowledge this if they are to mutually gain the most from their relationship with their medical mentors.
Bahner, David P; Adkins, Eric;	2012	How we use social media to	Demonstrate a supplement to a curriculum using ''push technology''	Type of study:	Year 4 Medical Students	Twenty-seven followers completed the survey with the	N.A.

Patel, Nilesh; Donley, Chad; Nagel Rollin, and Kman, Nicholas E.		supplement a novel curriculum in medical education	via Twitter and Facebook to deliver educational content to mobile devices.	Interventional Study/Pilot Twitter Ultrasound Educational Site<br><br>Methodology:<br>A curriculum consisting of high-yield ultrasound concepts was developed and posted to Twitter @EDUltrasound daily. Followers received tweets ‘‘pushed’’ directly to their mobile devices. Following the year-long program, followers were surveyed regarding the program’s effectiveness. To determine the ways in which tweets were reaching users, followers were categorized demographically.	Radiology Learning Supplement	majority (81.5%) being less than 30 years old. Many followers who completed the survey were medical students (63%), although interns (18.5%), residents (7.4%), and attendings (11.1%) also responded. Ultrasound experience ranged from novice (0–10 scans, 5.3% of respondents) to advanced (4100 scans, 47% of respondents). The majority of followers (55.6%)<br>were new to Twitter, although most followers (88.9%) found the Twitter feed user-friendly, the information useful (81.5%), and would like to follow more educational feeds via Twitter (59.2%). The Twitter app on mobile device was the most common way (80%) to access the information for the non-medical student followers, while medical students were more likely to use Facebook (35.3%) and Twitter (35.3%) via computer and also text messages and Facebook app.<br>Interestingly, the medical students (29.4%) were less likely (X 1⁄4 4.20, p5 0.04) than the non-students (70%) to have used Twitter	
Bellman	2003	Evaluation of a multi-professional mentoring scheme in primary health care	An 18-month qualitative evaluation of a multi-professional mentoring scheme has recently been completed (Bellman, 2002). The scheme, run by the Department of General Practice & Primary Care, Guy’s, King’s & St Thomas’s School of Medicine, aims to:<br>(1) encourage experienced individuals to share their knowledge and expertise in professional development<br>(2) provide support and encouragement between individuals in identifying professional development needs and creating a plan to meet those needs	Methodology:<br>Short Report of a multi-method data collection approach included reviews of documentation, exploratory interviews, participant observation, tracking the mentor/mentee process, field-work journals. Triangulation of the data and thematic analysis was undertaken.	Primary health care professional and administrative staff	The themes for both mentors and mentees encompassed:<br>. enlightening and empowering learning experiences<br>. demonstrable support for personal and professional challenges<br>. enhanced multi-professional working practices<br>. commitment to the evolving scheme.	N.A.

			(3) develop a multi-professional learning environment. The scheme enables primary health care professionals and administrative staff to engage together in personal and professional development.				
Blatt et al	2018	Preparing Interprofessional Faculty to Be Humanistic Mentors for Medical Students: The GW-Gold Mentor Development Program	The GW-Gold Humanistic Mentor Development Program addresses the challenge faced by medical schools to educate faculty to prepare students for humanistic practice. Grounded in Branch's Teaching Professional and Humanistic Values model, the program prepares interprofessional faculty mentoring teams in humanistic communities of practice. The teams consist of physician-psychosocial professional pairs, each mentoring a small student group in their professional development course. Through GW-Gold workshops, faculty mentors develop interprofessional humanistic communities of practice, preparing them to lead second such communities with their students. This article describes the program and its evaluation.	Methodology:<br>To assess outcomes and better understand the mentor experience, we used a mixed-method validating triangulation design consisting of simultaneous collection of quantitative (mentor and student surveys) and qualitative (open-ended survey questions and focus group) data. Data were analyzed in parallel and merged at the point of interpretation, allowing for triangulation and validation of outcomes.	Medical students	Mentors rated the program highly, gained confidence in their humanistic skills, and received high scores from students. Three themes emerged that validated program design, confirmed outcomes, and expanded on the mentor experience: (1) Interprofessional faculty communities developed through observation, collaboration, reflection, and dialogue; (2) Humanistic mentors created safe environments for student engagement; and (3) Engaging in interprofessional humanistic communities of practice expanded mentors' personal and professional identities.	• Outcomes support the value of the GW-Gold program's distinctive features in preparing faculty to sustain humanism in medical education: an interprofessional approach and small communities of practice built on humanistic values.
Bouchonville et al	2017	Endo echo improves primary care provider and community health worker self-efficacy in complex diabetes management in medically underserved communities	To determine whether participation in a multidisciplinary telementorship model of health-care delivery improves primary care provider (PCP) and community health worker (CHW) confidence in managing patients with complex diabetes in medically underserved regions.	Methodology:<br>A well-established healthcare delivery model, Project ECHO (Extension for Community Healthcare Outcomes) was applied to the management of complex diabetes (Endo ECHO) in medically underserved communities. A multidisciplinary team at Project ECHO connect-ed with PCPs and CHWs at 10 health centers across New	Primary care providers (PCP) and community health workers (CHW), community healthcare providers and individuals from the community	PCPs and CHWs in rural New Mexico report-ed significant improvement in self-efficacy in all measures of complex diabetes management, including PCP ability to serve as a local resource for other healthcare providers seeking assistance in diabetes care. Overall self-efficacy improved by 130% in CHWs (P<.0001) and by 60% in PCPs (P<.0001), with an overall large Cohen's effect size.	• Among PCPs and CHWS in rural, medically underserved communities, participation in Endo ECHO for 2 years significantly improved confidence in complex diabetes management. Application of the ECHO model to complex diabetes care may be useful in resource-poor communities with limited access to

				Mexico for weekly videoconferencing virtual clinics. Participating PCPs and CHWs presented de-identified patients and received best practice guidance and mentor-ship from Project ECHO specialists and network peers. A robust curriculum was developed around clinical prac-tice guidelines and presented by weekly didactics over the ECHO network. After 2 years of participation in Endo ECHO, PCPs and CHWs completed self-efficacy surveys comparing confidence in complex diabetes management to baseline.			diabetes specialist services.
Bramble et al	2018	Exploring and Improving Student Engagement in an Accelerated Undergraduate Nursing Program through a Mentoring Partnership: An Action Research Study	Similar to other distributed models, at the satellite campus, and students experience a sense of geographic, administrative, collegial and personal isolation from the main campus. A number of additional factors that contribute to the lack of engagement and poor sense of com- munity on the satellite campus include the accelerated nature of the degree programs to read, a predominance of culturally and linguistically diverse (CALD) backgrounds in the nursing cohort, a mix of blended and online programs and high sta attrition. // Managing the requirements for success in an accelerated program is particularly challenging for the high proportion of CALD students in the nursing cohort enrolled on campus, because, for most, English is their second language.	This Participatory Action Research (PAR) project aimed to engage students from an accelerated 'fast track' nursing program in a mentoring collaboration, using an interdisciplinary partnership intervention with a group of academics. Student participants represented the disciplines of nursing and paramedicine with a high propor- tion of culturally and linguistically diverse (CALD) students. Nine student mentors were recruited and	Student participants represented the disciplines of nursing and paramedicine with a high proportion of culturally and linguistically diverse (CALD) students.	Emergent themes were: 1) 'building relationships for active engagement', 2) 'voicing cultural and social hierarchies', and 3) 'enacting collegiate community'.	• The study offers insights into issues for contemporary accelerated course delivery with a diverse student population and highlights future strategies to foster an active student engagement.

				paired with academics for a three-month 'mentorship partnership' intervention. Data from two pre-intervention work-shops and a post-intervention workshop were coded in NVivo11 using thematic analysis. Drawing on social in-clusion theory, a qualitative analysis explored an iteration of themes across each action cycle.			
Cartledge, Peter; Miller, Michael, and Phillips, Bob	2013	The use of social-networking sites in medical education	A social-network site is a dedicated website or application which enables users to communicate with each other and share information, comments, messages, videos and images.	Type of study: Systematic Review<br><br>Methodology:<br>A systematic-review was undertaken using the PRISMA (Preferred Reporting Items for Systematic Reviews and Meta-Analyses) guidelines (Moher et al. 2009). Search strategy Using the methods described by Haig and Dozier (2003), the databases Medline, Embase (Appendix 2), PsycINFO, Educational Resources Information Centre (ERIC), CINAHL, the Cochrane library, the British Education Index (BIE) and the Research and Development Resource Base in Continuing Medical Education on the	Medical Education: Healthcare professionals and Medical Students	The search identified 1047 citations (Figure 1), and 77 articles<br>were reviewed in full. Six of the papers described using free web-based resources:<br>Facebook and Twitter. Using free web-based software should<br>be of interest to educators, especially those wanting to implement distance-learning modules in resource-poor countries. Three papers, by Bowen et al. (2012), Finlayson et al.(2010) and Keynejad et al. (2013), described the use of a custom-made website MedicineAfrica (http://www.medicineafrica.com/).<br>All papers, except Zolfo et al. (2010), described the use of<br>social-networking websites via personal computers. Zolfo<br>employed smart-phones to engage physicians in HIV clinics<br>in Peru.	• The interventions used in each paper were variable (heterogeneous) and because of the highly specific nature of the content, format and participants found in each would be difficult for other educators to know if using similar interventions in another environment with different students would be effective. However, together, these case-studies provide evidence of social-networking sites being used for educational purposes from four different continents and from two different disciplines for both undergraduate and postgraduate studies.

				Internet (RDRBWEB), were all searched from 01 January 2002 to 10 January 2012.			
Cheong, Clarissa Wei Shuen	2019	A systematic scoping review of ethical issues in mentoring in internal medicine, family medicine and academic medicine	To better understand and address mentoring abuse concerns, a systematic scoping review into prevailing accounts of ethical issues and professional lapses in mentoring is undertaken.	Type of study: Systematic scoping review<br><br>Methodology: Accounts of mentoring in internal medicine, family medicine and academic medicine in Pubmed, Embase, ERIC, ScienceDirect and Scopus databases were searched between 18th April and the 29th of June 2018. 98 articles were included in the final review.	Internal medicine, family medicine, academic medicine	Four areas of the mentoring process are particularly susceptible to ethical lapses. The first three are the matching process, evaluations of mentoring relationships and oversight and structuring of the mentoring process. All three practices draw attention to the host organization which is tasked to address issues with supporting and evaluating the matching, assessment, policing and structuring of the recruitment, matching, appraisal and support systems within the mentoring process.	• Unsatisfactory matching, misaligned expectations, inadequate mentor training, cursory codes of conduct, sketchy standards of practice, meagre oversight and unstructured processes have been identified as potential causes for ethical and professional breaches in mentoring practice.<br>• The host organization plays a major role in establishing codes of conduct, expectations, and holistically, longitudinally oversight of the mentoring process and mentoring relationships.
Chong, J. Y.	2020	Enhancing mentoring experiences through e-mentoring: a systematic scoping review of e-mentoring programs between 2000 and 2017	E-mentoring is proposed as a means of supplementing this dominant form of mentoring in medicine by providing accessible, timely and longitudinal support for mentees. However, with little is known about e-mentoring nor its role in a blended mentoring approach, a systematic scoping review is proposed to evaluate these gaps in understanding in order to better understand e-mentoring and assess the viability of employing e-mentoring practice to support novice mentoring	Type of study: Systematic Scoping Review<br><br>Methodology: Thematic analysis approach was used to thematically analyse accounts of e-mentoring across different settings. 6557 abstracts were identified, 109 full text articles were reviewed, and 18 articles were included and thematically analysed.	Medicine only	Thematic analysis of the 11 program reviews, 4 commentaries and 2 systematic reviews and 1 survey analysis included revealed 5 themes including characterization, role, process, platform, evaluation and relationships and blended approach	• The themes identified include definitions, role, stages, processes, platforms, evaluation, and relationships in e-mentoring. The themes identified provide a clinically relevant definition of e-mentoring, and in highlighting the similarities in the phases of novice and e-mentoring reaffirms the validity of a blended approach as a means of addressing shortfalls in mentoring in medicine/

							• Evidence presented by this systematic scoping review not only suggests that an e-mentoring approach is a distinct mentoring approach but one that is sufficiently complementary to novice mentoring to be used effectively within a blended approach.<br>• Overall data from this systematic scoping review suggests that an e -mentoring approach can enhance the mentoring experiences, support and outcomes of a novice mentoring program. Increased interactions provided by e-mentoring also help nurture better oversight of mentees and provide an additional means of overseeing individual mentoring relationships at a time when mentoring is increasingly under the microscope for potential abuse of mentoring processes and relationships.
Clark	2018	Why gerontology and geriatrics can teach us a lot about mentoring	Gerontology, geriatrics, and mentoring have a lot in common, for reasons found both in the origin of the term and in the application of aging-related concepts to understanding it.// More recently, the concept and importance of the role of mentoring in adult development were further developed and refined in the research of Levinson, Darrow, Klein, Levinson, and McKee (1978) for men	Type of study: Commentary<br><br>Methodology: N.A.	Gerontological and geriatrics educators	Though the topic of mentoring and its importance in higher education in particular are now studied and widely discussed (e.g., Johnson, 2007), gerontological and geriatrics education specifically can provide unique insights into the role of mentoring, based on some of the key concepts that emerge from the study of aging itself. These themes include the following: (1) the importance of interprofessional	• This discussion explores these topics and argues that gerontological and geriatrics educators have a particularly important role and responsibility in mentoring students, colleagues, and

			and Levinson and Levinson (1996) for women. Thus, the role of mentor as we now understand it has its meaning firmly rooted in an association with adult development, education, and wisdom.			leadership and modeling, (2) the application of the concept of "grand-generativity" to mentoring, (3) "it takes a community" to be effective in mentoring others, and (4) the need to tailor mentorship styles to the person and the situation.	administrators related to the very future of our field.
Coates, W. C.	2004	The virtual advisor program: linking students to mentors via the world wide web	The authors describe user characteristics of a Web based virtual advisor program that paired medical students with EM faculty advisors	Type of study: Quantitative<br><br>Methodology: Prospective users access the system from a link on the Society for Academic Emergency Medicine (SAEM) home page. On the initial visit, demographic information is collected.	Unspecified	Two hundred sixty-four students (183 males, 75 females, 6 unspecified) from North American (87) and international (25) medical schools requested a virtual advisor. One hundred twenty-one faculty advisors from 56 U.S. medical schools participated (86 [71%] males; 35 [29%] females). Students indicated reasons they sought a virtual advisor. Qualitative feedback was generally positive from advisors and advisees.	• Medical students at schools without affiliated EM residency programs may benefit the most from this program. Students who attend medical schools that do not mandate EM as part of the curriculum may not have access to board-certified emergency physicians with whom to discuss whether they are suited for a career in EM.<br>• Another significant issue is the lack of specialty-specific guidance for these students once they have identified EM as their targeted residency choice.
Ellaway, Rachel and Masters, Ken	2008	AMEE Guide 32: e-Learning in medical education Part 1: Learning, teaching and assessment	This Guide is presented both as an introduction to the novice, and as a resource to more experienced practitioners. It covers a wide range of topics, some in broad outline, and others in more detail. Each section is concluded with a brief 'Take Home Message' which serves as a short summary of the section. The Guide is divided into two parts. The first part introduces the basic concepts of e-learning, e-teaching, and e-assessment, and then focuses on the day–to-day issues of	Type of study: Recommended Guidelines<br><br>Methodology: N.A.	Medical Students, Doctors, Clinicians	N.A.	• In just a few years, e-learning has become part of the mainstream in medical education. While e-learning means many things to many people, at its heart it is about the educational uses of technology.<br>• Educational technologies can be used in support of

			e-learning, looking both at theoretical concepts and practical implementation issues. The second part examines technical, management, social, design and other broader issues in e-learning, and it ends with a review of emerging forms and directions in e-learning in medical education.				virtually any aspect of medical education.<br>• E-learning, e-teaching and e-assessment are related, but distinct areas of activity. Integrated e-learning systems in the form of virtual learning environments or learning management systems are now the norm.<br>• Working with online learners requires particular competencies and approaches of the tutor.<br>• Mobile learning affords many new opportunities to work with learners in new contexts.<br>• Some e-learning involves a focus on content while other forms focus on process.<br>• E-assessment presents particular challenges to both students and tutors.
Farnaz Heidari Clive Andrewes Kathleen Galvin Rebecca Pendlebury Iain Graham	2002	Shared learning and mentoring for newly qualified staff: Support and education using an interprofessional approach	The aim of this evaluation was to explore the perceived benefit of interprofessional mentorship and learning for newly qualified nurses and<br>PRHOs. The objectives, therefore, were to:<br>- Examine the experiences of mentoring by a more senior member of<br>staff on stress and development of junior staff<br>- Examine staff experiences and	Type of study: Interventional study with mixed methods<br><br>Methodology:<br>An ethnographic research approach was used, and data collection involved one-to-one interviews, self-completed questionnaires and observation.	Unspecified	The shared mentoring and learning was well received by all Trusts as the Scheme rolled out. The shared mentoring component was more positively<br>evaluated by all participants and utilised by the PRHOs than the shared learning.	Some ideas for improving shared learning:<br>• Find appropriate time for all staff, for example, avoiding lunchtime hand over for nurses<br>• Incorporating the shared learning sessions as part of the educational

			perceptions of shared learning<br>- Explore professionals' understanding of interprofessional working and shared learning<br>- Identify factors which support and hinder collaboration, co-operation and learning among nurses and doctors<br>- Develop concepts to promote interprofessional working (including mentoring) and shared learning.	These were conducted before the Scheme started and repeated after six months. Any documents such as evaluation forms generated from the study were also examined. Questionnaire designs used were Parsell and Bligh's (1999) Readiness for Interprofessional Learning, Brown et al.'s (1986) Group Identification Scale, and Carpenter's (1995) Professional Stereotypes. The sampling was purposive and the criteria were explicit and systematic as Hammersley and Atkinson (1995) suggest. For the questionnaires, all mentors, mentees, Project Leads and Clinical Tutors took part (N=141). A simple random sampling was used to select the participants for one-to-one interviews (N=34) and they needed to meet the following criteria: Senior member of staff (at least two years of clinical experience) Newly qualified nurses and PRHOs Clinical Tutors and Post graduate Managers Have an interest in			requirements for both professions<br>• Giving protected time to the shared learning sessions as part of their educational requirement<br>• Informal sessions at directorate level<br>• Further training for mentors to identify learning opportunities for newly qualified staff during day to day working in clinical settings<br>• Identification of individual specialists from all professional groups willing to facilitate shared learning sessions who also have the skills to work across different professions<br>• More training for facilitators in conducting the sessions. Bournemouth University to develop a training package to help facilitators with shared learning sessions<br>• Various professions getting together to work on a project of common interest, each learning about other professions – not in a lecture theatre, would need a more creative approach

				interprofessional learning and working Voluntary participation Willing to take part in the shared mentoring and learning programmes.			• The use of problem-based learning or case study approach<br>• Deaneries to encourage Clinical Tutor and Postgraduate Medical Centre to provide opportunities for the Core Curriculum to be delivered using a shared learning approach.
Forgie, Sarah Edith; Duff, Jon P, and Ross, Shelley	2012	Twelve tips for using Twitter as a learning tool in medical education	Describe how to improve the effectiveness of web-based multimedia instruction for clinical learners. Web-based multimedia instruction (WBMI) provides learners with self-directed independent learning opportunities based on didactic material enhanced with multimedia features such as video and animations. WBMI may be used to replace other didactic events (e.g. lectures) or it may be provided in addition to other learning opportunities.	Type of study: Literature review<br><br>Methodology: We derive our tips based on the literature and on our experiences creating WBMI materials and studying their effectiveness and implementation in surgical clerkships across multiple medical schools. We explored the impact of evidence informed WBMI module design features on knowledge building, clinical reasoning, satisfaction, and motivation in a randomized controlled trial at 7 U.S. medical schools. We also conducted a qualitative study to examine how unique environmental and cultural factors at the different medical schools might impact educational effectiveness of the WBMI modules in	Healthcare Professionals and trainees	The following 12 tips have been developed to help guide faculty through some of the key features of the effective use of WBMI in clinical teaching programs. These tips are based on more than a decade developing, using and appraising WBMI in support of surgical clerkship education across the USA and beyond and they are intended both to inform individual uses of WBMI in clinical training and to guide the strategic use of WBMI in clinical clerkship curricula.	• Twitter is a relatively new social medium, and its use in higher education is in its infancy. With further research and thoughtful application of media literacy, Twitter is likely to become a useful adjunct for more personalized teaching and learning in medical education.

				parallel to this randomized control.			
Frei, Esther	2010	Mentoring programs for medical students-a review of the PubMed literature 2000-2008	Although mentoring is acknowledged as a key to successful and satisfying careers in medicine, formal mentoring programs for medical students are lacking in most countries. Within the framework of planning a mentoring program for medical students at Zurich University, an investigation was carried out into what types of programs exist, what the objectives pursued by such programs are, and what effects are reported.	Type of study: Review<br><br>Methodology:<br>A PubMed literature search was conducted for 2000 - 2008 using the following keywords or their combinations:<br>mentoring, mentoring program, medical student, mentor, mentee, protégé, mentorship. Although a total of 438 publications were identified, only 25 papers met the selection criteria for structured programs and student<br>mentoring surveys.	Medical students as mentees	The mentoring programs reported in 14 papers aim to provide career counselling, develop professionalism, increase students' interest in research, and support them in their personal growth. The 11 surveys address the requirements for being an effective mentor as well as a successful mentee.	• Evidence from the reviewed papers shows that three factors are important for effective mentoring programs. Firstly, for students pursuing an academic career, a one to-one mentorship with an advanced scientist involving the junior in his/her research proves most effective.<br>• Secondly, the mentor must serve as both a professional and personal role model.<br>• Thirdly, provision of career counselling by mentors leads to juniors' making an earlier choice in terms of specialty and career<br>• Most conceptual and survey papers focus on the qualities required to become an effective mentor. A confidential relationship and the mentor's commitment to his/her mentee's professional and personal development are considered to be the main requirements
Gill, Peter J	2019	Building capacity in evidence-based medicine in low-income and middle-income countries: problems and potential solutions	Achieving improvements in healthcare globally<br>requires building and sustaining early and mid-career<br>researchers (EMCRs). Yet, there are big gaps<br>in both critical appraisal and research capacity,<br>particularly in low-income<br>and middle-income	Type of study: Opinion<br><br>Methodology: N.A.	Unspecified	EMCRs seeking a career in EBM face numerous challenges, particularly in LMICs	• To close this gap, sustainable regional capacity building is needed through targeted mentorship for EMCRs. Networked models and linkage systems could connect EMCRs in LMICs with suitable mentors locally and in high-

			countries (LMICs), and this hinders development in these regions.				income countries through online mentoring to support the development of critical appraisal and research capacity in evidence-based healthcare
Goff et al	2017	Impact of a national antimicrobial stewardship mentoring program: Insights and lessons learned	The impact of an interprofessional mentoring program to ad- vance antimicrobial stewardship programs (ASPs) in selected U.S. hospi- tals and lessons learned are described. //The literature in the elds of business, education, and medicine supports thevalueofmentoring;however,lit- erature on antimicrobial stewardship and mentoring is scarce. As stated by Crosby,10 "Mentoring is a brain to pick, an ear to listen, and a push in the right direction."	Methodology: A seven-step mentoring process with self-assessment, tele- phone calls, continuing education, a one-day onsite visit, action plan, and outcome data collection and analysis was provided to ASP teams at nine hospitals. Six hospitals completed the program.	Hospital administrators, pharmacy directors, infectious diseases physician and pharmacists	A significant improvement in the timeliness and appropriateness of i.v. antibiotic therapy (de ned as a hang time within one hour after prescriber order entry and broad- spectrum coverage for gram-negative pathogens administered rst when combination therapy was used) was observed in patients with sepsis over the 12-month period after implementation of the mentoring program.	• A mentoring program for antimicrobial stewardship provided the perspective that comes from experience. Engagement of hospital ad- ministration was a key factor for both developing and sustaining a stewardship program.
Gray, Kathleen; Annabell, Lucas, and Kennedy, Gregor	2010	Medical students' use of Facebook to support learning: Insights from four case studies	Educators are becoming increasingly interested in how online social networking software can be harnessed for educational purposes. There is empirical evidence that social networking sites are popular among university students and that students are using social networking sites specifically in connection with their studies. Facebook's features could provide useful support for student collaboration, student-generated content, student–student communication and the personalisation and socialisation of student work.	Type of study: Retrospective review of how Facebook is used by medical students for their learning<br><br>Methodology: Mixed methods research using a survey and case studies was conducted in the undergraduate medical school of a large metropolitan university at the end of 2008. The questionnaire was modelled on related surveys (Conole et al. 2008; Ipsos MORI 2008; Sandars et al. 2008). It comprised 14 items regarding demographics,	Undergraduate students from years 1,2,5,6	The majority of students had used Facebook (87.0%, n 1⁄4 759). Most accessed it weekly or more often (90.5%) and over half (55.2%) daily. One in four students had used Facebook for educational reasons (25.5%, n 1⁄4 660). Of the 496 students who said that they had not done so, half indicated that they would consider using it for this purpose (50.0%). The combination of participants who had used Facebook educationally and those who would consider using it was 64.7% of Facebook users, or 54.5% of the total sample. Over one-quarter of students who had used Facebook for educational reasons had used the Facebook 'groups' feature specifically (27.7%).	• The majority of students had used Facebook, with substantial numbers of medical students are giving time and attention to using Facebook for study purposes. The precise extent of use is still unclear as there was a disparity between the educational use that students reported in the survey and that which was observed in the case studies, even allowing for overlapping group membership.<br>• These case studies showed a variety of rationales and aims,

				frequency and extent of Facebook use and experiences and perceptions of Facebook use for educational purposes. The latter questions gave examples such as for discussion, sharing resources or giving or getting help about coursework, clinical cases, assessment and student administration matters. Three semi-structured one-hour interviews were conducted, each involving two students.<br>The analytical framework was used as a template for describing and comparing cases. One researcher did initial data analysis and another reviewed the analysis to strengthen consistency and reliability.			not unlike those that might underlie any informal in-person study group, for students' establishment of Facebook study groups.<br>• Understanding the contexts that give rise to them and their ambitions may give educators insights that are relevant to improving aspects of course structure or delivery.<br>• In particular, in light of disparaging comments that emerged during this investigation, educators interested in using online learning to enhance a course may need to review their current implementations of LMSs.
Griffiths, M	2005	E-mentoring: does it have a place in medicine?	Over the past decade there<br>has been a substantial increase in work into e-learning support, such as e-moderating, and other mentoring and support systems such as online communities of practice. It is not the intention here to explore these literatures<br>but to concentrate specifically on one area e-mentoring.	Type of study: Opinion<br><br>Methodology: N.A.	Unspecified	N.A.	• Many educators and organisations (including the medical profession) remain suspect about the new and growing field of e-mentoring. However, research by Preece and her colleagues has shown how the internet can be used to express empathy, particularly in online patient support communities. Furthermore, Griffiths has argued that online

							relationships can be just as real and intense as those in the offline world, and there should be little surprise that psychologists and educators are beginning to establish online therapeutic relationships.<br>• Through examination of the literature, e-mentoring seems to be advantageous for a number of reasons. Furthermore, all the advantages of e-mentoring are equally applicable in medical settings<br>• However, e-mentoring may not appropriate for everyone and those participating should at the very least be comfortable in expressing themselves through the written word. As with any new frontier, there are some issues to consider before trying it. E-mentoring also provides
Hall et al	2009	Linking Health Professional Learners and Health Care Workers on Action-Based Improvement Teams	Medical students, nursing students, and other health care professionals in training were integrated with health care workers on interprofessional quality improvement (QI) teams at our academic health center. Teams received training in QI, accompanied by expert QI mentoring, with dual goals of increasing expertise in improvement while improving care.	Type of study: Interventional study<br><br>Methodology: Eighty-six learners and health system workers participated in 12 improvement teams in 2 years.	Medical students, nursing students, and other health care professionals	Upon completion of the training, participants expressed that the program enhanced QI and teamwork skills and increased understanding of other health care professions. At the end of the program, fourth-year medical students showed greater ability to apply QI skills, as measured by the QI Knowledge Assessment Tool than did control students who did not participate in the program (P < .0001 in 2006–	• The design of "learning QI by doing," accompanied by just-in-time training and ongoing expert mentoring in QI, was identified by faculty as the most important factor contributing to success.

						2007 and P< .0005 in 2007–2008). Many teams were successful in improving care processes.	• This model successfully improved application of QI skills by learners while improving care within our academic health center. Testing of the model at other academic health centers and in other training environments is warranted.<br>• Participants strongly expressed that the process facilitated in the learning of QI skills, team- work, and greater appreciation for other health care professions.<br>• The value of centering the learning about improvement within a meaningful QI project was highlighted by participants. Participants felt strongly that peers would benefit from participating in similar programs. In both years, there was a demonstrable improvement in QI proficiency as measured by the QIKAT scores when comparing medical student participants with control participants.
Haralampos M. Moutsopoulos	2019	Mentoring in medicine	N.A.	Type of study: Editorial	Unspecific	N.A.	• Traditional mentoring has been redefined and it is partially— or

				Methodology: N.A.			entirely in some disciplines—sought and offered through digital communication<br>• E-mentoring provides learning, advising, encouraging, promoting and modelling, that is unrestricted, without physical and geographical boundaries, yet qualitatively different than face-to face mentoring.<br>• One could argue that e-mentoring within medical education complements and extends what is achieved by traditional mentoring in the sense that "e-mentors" can provide immediate response and feedback on theoretical medical/ clinical issues and career advice, however since medical education requires a hands-on training, patient-doctor interaction and empathy, it is highly unlikely that this in-person experience will be fully substituted by technology and traditional mentoring will continue its valuable role and effect.
Hartnup, Becky; Dong, Lin, and Eisingerich, Andreas Benedikt	2018	How an Environment of Stress and Social Risk Shapes Student	Critical gaps remain in our understanding of what drives students to actively engage with social media and the barriers that impede such engagement in the phase of university	Type of study: Qualitative and Thematic Analysis of In-Depth Interviews	First Year Medical Students	N.A.	• Maintaining existing relationships, building new relationships, and seeking academic support drove students

		Engagement With Social Media as Potential Digital Learning Platforms: Qualitative Study	adjustment. The lack of understanding prevents us from maximizing the benefits that social media can provide for students, such as creating new opportunities for learning and enhancing learning efficiency.				to engage with social media and use it for learning.<br>• The key inhibitors that emerged from the data were (1) collapsed online identity, (2) unclear and even conflicting norms, (3) the desire to present an ideal self, and (4) perceived academic competition within their social groups.<br>• The findings also highlight that students engaged with various social media platforms in different ways.
Heather Pearce, David Blainey	1999	Nurse mentors for preregistration house officers	Newly qualified junior doctors experience 'reality shock' when they start work on the wards. This article describes the development of a nurse mentorship scheme which uses the skills and expertise of senior ward nurses to help them through this transition. It has proved to be both popular and successful.	Methodology:<br>The nurse mentorship scheme offered a mechanism to assist in the transition from student to doctor, provided a structure for enhanced learning and support for these skills, and could be taught well by senior nurses.<br><br>The pilot study was carried out on two general medical wards and ran for two 6-month periods covering the first and second placements of the PRHO. It included 4 PRHOs, 2 nurse mentors and 2 consultants.	Medicine only	Both nurses and preregistration house officers felt they had benefited from the scheme with greater understanding of each other's roles and responsibilities.<br><br>A nurse mentorship scheme can enhance team working at ward level.	The main advantages for the participants were identified as:<br><br>PRHO<br>• The PRHO had an established point of contact at ward level, thus ensuring that they felt valued and cared for<br>• Nurses were able to teach clinical and non-clinical skills in an effective way Team working was improved<br>• The PRHO developed a greater understanding of the role of others within the team.<br><br>Nurse<br>• Nurses felt their skills and knowledge were being recognized

							• Nurse mentors felt they had a greater responsibility for the development of the PRHOs clinical skills which enhanced the working relationship<br>• Nurse mentors felt that they were able to offer a fairer evaluation of the PRHOs abilities.<br><br>The pilot study also identified some problems:<br>• Time (as usual) was limited<br>• The unpredictable nature of ward work prevented the mentors and PRHO getting together as often as they would have liked.<br>• For the pilot project the ward sisters and the consultants already worked closely together, and all had been involved in the project from early on. Even so, we considered that the project and the results could be transferred to other areas, and it became clear that the other PRHOs wanted to take part.
Heidari	2007	Interprofessional mentoring - Exploration of support and professional	The aim of this study was to examine whether newly qualified healthcare staff<br>can be supported in their journey to become a practitioner using an	Methodology: Interventional study with mixed method approach (an ethnographic	Newly qualified doctors (pre-registration house officers - PRHOs)	All participants were in favour of interprofessional education, which they believed would aid collaborative working and improve the practice environment. It was acknowledged that	• This study has demonstrated that with adequate funding, long-term plans (broken up into

		development for newly qualified staff	interprofessional framework to mentoring. The objectives, pursuant to the aim, were to:<br>• Examine the experiences of interprofessional mentoring for mentors, PRHOs and those involved in implementing the scheme;<br>• Identify factors that support and hinder interprofessional mentoring among nurses and doctors;<br>• Identify any benefits of interprofessional mentoring for the learning path of nurses and doctors;<br>• Explore perceived benefits for healthcare delivery.	approach, which allows the use of both qualitative and quantitative methods of data collection, was adopted. Data collection methods include: interviews, questionnaires, observations)	mentored by senior nurses	nurses and doctors have different priorities and learning needs. Support in the clinical area and having someone to talk to about fears, worries and practical problems were viewed as most important by newly-qualified doctors who all expressed similar anxieties. It was evident from the interviews that support for junior staff comes from a variety of sources and is dependent on the experience and personality of the individual providing the support. Mentoring different professional groups was found to be a positive experience by most participants. Mentoring was used to varying degrees by junior staff depending on their needs and on their own personal views and attitude towards the initiative. Collaboration was seen as the main focus of interprofessional working.	manageable short term goals), and the support of management, and senior and junior staff, interprofessional mentoring can be a viable approach to supporting newly qualified staff.<br>• Furthermore, it can shed light on our understanding of the roles and contributions of other staff, improve communication and collaboration, and ultimately, through influencing the care received by patients, improve staff job satisfaction for staff and a more efficient use of resources.
Hodgson, J. C. and P. Hagan	2020	Medical Education Adaptations During a Pandemic: Transitioning to Virtual Student Support	The sudden introduction of social distancing measures designed to stem the spread of the virus resulted in a national switch to online teaching and removed the ability to provide students with support in-person.<br>The challenge for staff was to ensure student welfare and wellbeing were maintained as much as possible, but also track any students who needed additional pastoral support, all whilst being unable to access campus services in the usual way. Using the existing system of Personal Tutor groups, Personal Tutors were encouraged to switch to online virtual tutor groups via University supported software packages, which all staff and students have access to free of charge. Both universities have institutional subscriptions to Microsoft TeamsTM	Type of Study: Interventional<br><br>Methodology:<br>Using the existing system of Personal Tutor groups, Personal Tutors were encouraged to switch to online virtual tutor groups via University supported software packages, which all staff and students have access to free of charge. Both universities have institutional subscriptions to Microsoft TeamsTM application, which was used for 1:1 video calls	Medical students		• Students adopted the model very readily, and with simple guidance about using the platform, found it convenient to use on their smartphone or computer. Many staff hadn’t considered engaging with students in this way, but the software provided an accessible means of bridging the instant messaging/formal meeting gap.<br>• Video calls, rather than just audio calls, improved the quality of the experience for both

			application, which was used for 1:1 video calls and for the instant messaging group chat function.	and for the instant messaging			parties. The scheduled video calls also provided structured points for social contact, which proved important for maintaining routine, supporting wellbeing and providing a sense of perspective and connection to the course.<br><br>• Feedback about the group chat function was also positive, with students appreciating the connection with other members of their cohort.
Hollinderbäumer, Anke; Hartz, Tobias, and Ückert Frank	2013	Education 2.0 - How has social media and Web 2.0 been integrated into medical education? A systematical literature review	Web 2.0 technologies are increasing. These tools and communication technologies are used by students, instructors and patients in multiple ways to gain information and to communicate. Therefore, these tools should also be assimilated into the education of future doctors. Universities that integrate these technologies into teaching have been able to show that their students are more motivated and discuss issues in a more lively fashion. Thus, the inclusion of Web 2.0 tools and social media takes into account the changes to communication and information channels and conveys the necessary skills.	Type of study:<br>Systemic Review of use of Web 2.0 in Medical Education<br><br>Methodology:<br>A systematic literature review based on the research question was conducted on 1 May 2012, using PubMed. In the search query: 16 terms (MeSH) related to the term "medical", 43 terms (MeSH) related to the term "education" and 51 terms (MeSH) related to the term "social media" were used. By connecting these terms, 1245 results were obtained. This	Medical students	N.A.	• In the literature from the first 2 years (2007/2008), general user behaviour was described, and requirements derived from the use of new media in medical education were proposed. The publications in 2009 begin to put thought into negative effects of using social media in the educational context. These critical discussions continue in the following years (2010/2011). Studies appear on the use of concrete Web 2.0 tools

				was reduced to 84 results after applying the limiting factors and the appropriate inclusion and exclusion criteria. In the subsequent examination of the abstracts, articles dealing with health care provision, and educating and informing the patient were excluded. The remaining publications were evaluated in their full-text form. At this point, two additional articles were excluded. One was related to training librarians, and the other was about the Greek post-secondary education system. Therefore, 20 articles were examined in detail.			in special teaching and learning forms, for example in problem-oriented learning.
Hossain, Ibtesham Tausif; Mughal, Umair; Atalla, Bashar; Franka, Mustafa; Siddiqui, Sarim, and Muntasir, Mohammed	2015	Instant messaging – one solution to doctor–student communication?	In the last decade, social media has infiltrated into many dimensions of life forming the fabric of communication and innovation. Many hospitals still use outdated pager systems as the foundation for clinical communication. From a medical student's perspective, barriers to communication with supervisors present unique obstacles to learning. Clinical clerkship is largely unstructured with topics determined by presenting conditions of patients at the bedside and interests of the teacher. This has contributed to little consistency in delivery of the core undergraduate syllabus. It is further compounded by the fact that this model of opportunistic teaching naturally relies heavily on communication between students	Type of study: Letter to the editor<br><br>Methodology: N.A.	Clinical medical students	N.A.	• The scope of instant messaging is well known but has yet to penetrate the healthcare environment formally. Capitalising on this widely used technology can aid in the communication and ultimately learning experience of medical students. The wider potential of such technology is exciting and can provide an alternative mode of clinical communication between surgical and

			and teachers, and currently there is little in the way of an effective system in place to foster a conducive and productive experience.				medical teams with the possibility of a purpose-built instant messaging service for healthcare professionals going beyond the advantages of medical education.
Hunter, J. J., et al.	2008	A novel network for mentoring family physicians on mental health issues using e-mail	Family practitioners are significant providers of men-<br>tal health care and routinely report difficulty acquiring timely support in this area. The Collaborative Mental Health Care Network assembled groups of family practitioners and provided them with mental health practitioner mentors. This article addresses communication in the Network, its effect on family practitioners, and the role e-mail plays.	Type of study: Descriptive study<br><br>Methodology:<br>This descriptive study utilizes two sources of data: a<br>quality assurance survey administered to family practitioners in the Network and a sampling of e-mail correspondence between<br>family practitioners and mental health and addiction mentors, examined qualitatively.	Family practitioners and mental health practitioners	Family practitioners in the Network requested consul-<br>tation on pharmacotherapy (53%), psychotherapy (34%), treat-<br>ment review (27%), and diagnosis (24%). Satisfaction with the<br><br>Network was high, with 88% of family practitioners reporting an improvement in ability to provide mental health care.	• E-mail analysis suggests that mentors convey information directly and indirectly and that a knowledge hierarchy, but not a power hierarchy, develops. The trusted relationship between the mentee and mentor is an important context for effective education.<br>• This model of mentoring is highly satisfactory to family practitioners and correlates with increased confidence in caring for patients with mental health issues. E-mail is a promising strategy for effective feedback and support between family practitioners and specialists.
J. James Cotter et al	2010	Designing a multi-disciplinary geriatrics health professional mentoring program	This paper describes a Geriatric Health Professionals Mentoring Program designed to address recruitment and retention of health professionals in geriatrics and gerontology.	Type of study:<br>Interventional study<br><br>Methodology:<br>The training provided information on the mentoring process, negotiating mentoring agreements, and coaching mentees. The	Health professionals in geriatrics and gerontology	Trained mentors expressed satisfaction with the program and were comfortable about general mentoring but less confident about specific guidance for mentees.	• A cadre of mentors can be successfully trained to assist geriatric health professionals to better care for older Americans.

				evaluative frame-work described examines: (a) the effects of mentoring, (b) reactions of mentors and mentees, and (c) the effect of intervening variables.			
Jaffer, U., Vaughan-Huxley, E., Standfield, N., & John, N. W.	2013	Medical Mentoring Via the Evolving World Wide Web	The World Wide Web (WWW) has evolved, as a technology, to become more interactive and person centric, tailoring itself to the individual needs of the user. This changing technology may open new avenues to foster mentoring in medicine.	Type of study: Systematic Review<br><br>Methodology:<br>A search of the MEDLINE database from 1950 to 2012 using the PubMed interface, combined with manual cross-referencing was performed using the following strategy: (“mentors”[MeSH Terms] OR “mentors”[All Fields] OR “mentor”[All Fields]) AND (“internet”[MeSH Terms] OR “internet”[All Fields]) AND (“medicine”[MeSH Terms] OR “medicine”[All Fields]) AND (“humans”[MeSH Terms] AND English[lang]). Abstracts were screened for relevance (UJ) to the topic; eligibility for inclusion was simply on screening for relevance to online mentoring and web-based technologies.	Medicine and Surgery	Forty-five papers were found, of which 16 were relevant. All studies were observational in nature. To date, all medical mentoring applications utilizing the World Wide Web have enjoyed some success limited by Web 1.0 and 2.0 technologies.	• With the evolution of the WWW through 1.0, 2.0 and 3.0 generations, the potential for meaningful tele- and distance mentoring has greatly improved.

Jalali, Alireza; Sherbino, Jonathan; Frank, Jason, and Sutherland, Stephanie	2015	Social media and medical education: Exploring the potential of Twitter as a learning tool	The American Society of Nephrology (ASN) has turned to social media, namely Twitter, to uses its annual conference to inform and educate the public about kidney disease. Since the dissemination of information is necessary if Twitter is to be considered a tool to increase public awareness of kidney disease, a study was undertaken to analyse tweets during the official 2011 Kidney Week conference. Through linguistic content analysis, the authors found that Twitter can be used to disseminate educational information about kidney disease if the tweets had three key features: informative content, internal citations, and a positive sentiment score (calculated through content analysis of each tweet).	Type of study: Qualitative Analysis (Grounded Theory)<br><br>Methodology:<br>For this study we sought to establish Twitter as a learning tool within the psychological literature before turning our attention to the creation, use, and modification of typologies as a further frame for this programme of research. Qualitative coding methods were applied to the #ICRE2013 Twitter dataset.	Unspecified	Among the 373 ICRE 2013 conference participants, there were 4,958 tweets during the conference (26 – 28 September 2013). This resulted in an average of 34 tweets per hour with 4,815,052 impressions.	• The large majority of healthcare conferences have spikes in tweet activity during conference days. Regarding cognitive dimensions of learning that resulted from the Twitter activity, Tweets were analyzed according to application of the 'How People Learn' Framework, which includes Preconception, Framework, Metacognition/ Reflection. Twitter can be used as an influence generator in creating waves of influence through original posts and selective re-tweeting at a medical conference. These leaders contribute heavily to the community, and they contribute to the critical mass of the conversation, but more importantly the community can rely on these individuals to keep the conversation going and increase the robustness of the ongoing activity.
Jana Lait a,*, Esther Suter a,1, Nancy Arthur b,2, Siegrid Deutschlander a,3	2011	Interprofessional mentoring: Enhancing students' clinical learning	This intervention was part of a project funded by Health Canada's Interprofessional Education for Collaborative Patient-Centred Practice initiative, in which we	Type of study: Interventional study with qualitative methods (exploratory group and individual	Unspecified	in IP mentoring, 1) provider commitment was important, 2) the activities students engaged in varied in complexity, and, 3) students	• IP mentoring added to students' clinical experiences without reducing their learning

			piloted strategies to increase IP capacity in health providers and students in clinical sites in Alberta, a western province in Canada. The focus of this paper is the strategy of providing students with IP learning in their clinical placements through IP mentoring, and perceived benefits for students.	semi-structured interviews for evaluation)		had positive learning outcomes.	of discipline-specific skills.<br>• For academia, the advantage of an IP mentoring approach is that it does not require major curricula changes.<br>• For practice, the advantage of IP mentoring is that it does not require substantive changes to the work of healthcare providers at students' practicum sites; providers simply act as mentors in an informal fashion with a small time commitment.
Jiwa, A., Fardanesh, A., Stavropoulou-Tatla, S.	2019	Comment on: Medical student mistreatment by patients in the clinical environment–a student perspective	To offer anecdotal evidence of low-level reporting regarding medical student mistreatment.<br><br>To offer interventions to tackle the issue of mistreatment.	Type of study:<br>Letter to Editor<br><br>Methodology:<br>Not explicitly stated though it would appear that this is built upon anecdotal evidence and a literature review.	Medical students	N.A.	• Mistreatment in the hospital setting occurs due to a culture of tolerance, a lack of awareness of the reporting process and a reluctance to give feedback to staff<br>• Interventions to overcome this include implementing a discussion group, online forums, e-mentoring and giving seminars on identifying mistreatment and emphasising the importance of reporting.
John B.A.	2012	Kinship-King's Social	At a round-table discussion at the Brescia Colloquium 2011, the need	Type of study:	Undergraduate medical students	The site has initially been tested by 650, self selected medical students for	• Kinship could play a major role in keeping

		Harmonisation Project. Pilot phase of a social network for use in Higher Education (HE)	for a university hosted, members only social network was discussed and explored. Benefits were considered to include enhanced communication, collaboration, sense of community, informal learning opportunities and enhanced skills for the establishment of an appropriate online professional profile. There are already a number of established exemplars of open source social networking platforms for use in education. Therefore evaluation of the current popular functionality and systems being utilised by other universities such as Brighton, Leeds, Birmingham, Hong Kong and Harvard (all currently use an open source platform called Elgg) was considered valuable in order to inform the selection and customisation of an appropriate social networking platform for the College.	Interventional Study/Pilot Social Network Site<br><br>Methodology:<br>An internal eLearning content developer liaised with two specialist contractors to create templates for the site, respecting College policy, regulations and guidelines. The site uses an academic URL and was granted access to the university Active Directory to provide simple login using the standard institutional username and password. It is intended to circulate questionnaires and run formal focus groups.		three months and their views were sought at a series of in-formal focus groups in order to eliminate bugs and inform the initial incarnation and functionality of the site.	alumni in touch with the university, acting as mentors and inspiration to prospective students (who can be invited in), the site will function as an online yearbook, a place to collaborate and communicate around assignments and university based social activities and events, a place to model digital professionalism and even potentially as a notice board for events, a place to advertise the sale of books and the rental of accommodation.
Kalet, Adina L; Sanger, Joseph; Chase, Julie; Keller, Allen; Schwartz, Mark D; Fishman, Miriam L; Garfall, Alfred L, and Kitay, Alison	2007	Promoting professionalism through an online professional development portfolio: successes, joys, and frustrations	Medical educators strive to promote the development of a sound professional identity in learners, yet it is challenging to design, implement, and sustain fair and meaningful assessments of professionalism to accomplish this goal.	Type of study: Perspective<br><br>Methodology:<br>The authors developed and implemented a program built around a Web-based Professional Development Portfolio (PDP) to assess and document professional development in medical students at New York University School of Medicine.	Undergraduate pre-clinical and clinical medical students	A majority found creating the portfolio challenging. Revealing a significant opportunity for improvement, a majority found their faculty mentors unprepared for the end-of-year meeting. There were no significant differences in survey responses between the classes, and there were no differences in survey responses between the 48% (79/164) of students who reported experiencing significant technical problems with the PDP and those who did not.	• A Web-based PDP promoted self-regulation on an individual level because it facilitated narrative reflection, self-assessment, and goal setting, and it structured mentorship. Therefore, the PDP may prepare students for the self-regulation of the medical profession—a privilege and obligation under the physician's social contract with society.

Kamin, Carol; Glicken, Anita; Hall, Michael; Quarantillo, Barb, and Merenstein, Gerald	2001	Evaluation of Electronic Discussion Groups as a Teaching/Learning Strategy in an Evidence-based Medicine Course: A Pilot Study	Computer-mediated communication (CMC) reflects the ability of learners to participate in online discussion groups, expressing their own ideas and viewpoints and collaborating with their teachers and peers in a true interactive learning environment. Many studies have explored the advantages of computer-mediated communication in a variety of academic settings mostly in undergraduate courses. The most frequently mentioned benefit noted in these studies of online discussion groups was the aspect of asynchronicity, which freed students from the limitations of both time and distance. This convenience allowed students to learn at their own pace, with more time for reflection and formulation of responses.	Type of study: Interventional Pilot Study<br><br>Methodology:<br>We developed a CMC component for an Evidence-based Medicine (EBM) course for second-year physician assistant students at the University of Colorado Health Sciences Center. Upon completion of the course, we evaluated the effectiveness of this new strategy by examining student performance and attitudes based on discussion group type, CMC or face-to-face. This<br>process is facilitated by small mentor groups, in which students share their insights and opinions, and receive feedback from their peers and the faculty facilitator.	2nd Year Physician Assistant Students	N.A.	• Group assignment did not impact student performance on the final examination. All students, regardless of group assignment, significantly improved their post-test scores over the pre-test scores, but in spite of this difference, student performance in this group was not adversely affected. Student attitudes on issues related to the course, the use of technology for delivery, discussion, and skill acquisition were overwhelmingly comparable between group types.<br>• Attitudinal differences were found to exist with respect to: participation in discussions about course material with the face-to-face groups reporting a greater participation rate.
Ken Masters, and Rachel Ellaway	2009	e-Learning in medical education Guide 32 Part 2: Technology, management and design	With e-learning now part of the medical education mainstream, both educational and practical technical and informatics skills have become an essential part of the medical teacher's portfolio. The Guide is intended to help teachers develop their skills in working in the new online educational environments, and to ensure that they appreciate the wider changes and developments that	Type of study: AMEE Guide<br><br>Methodology: N.A.	N.A.	N.A.	• E-learning involves many dimensions in addition to its educational impact. Political, psychological, legal and ethical issues all need to be considered. Assessing the value of e-learning requires a range of different economic analyses.

			accompany this ‘information revolution’.				• The design of e-learning is a fundamental determinant on its success. This includes user interface design, accessibility and domain alignment. Research and development is an essential and ongoing aspect of e-learning practice.<br>• Healthcare education informatics affords better use and understanding of the many issues and themes around information systems in healthcare education.
Kim E.J.	2019	Working effectively with long-distance mentors	To draw from the author’s experience to outline how to build and maintain effective long-distance mentoring relationships.	Type of study: Editorial<br><br>Methodology:<br>Not explicitly stated though it would appear that this is an editorial built upon a literature review.	Dermatology	N.A.	• Planning and committing to a regular schedule of communication is key to building the relationship.<br>• It is important to agree upon the goals and framework of the relationship from the beginning.<br>• Other ways to maximise the relationship is by being an active listener, to have spontaneous communication and be committed.
Kind, Terry; Patel, Pradip D.; Lie, Désirée, and	2013	Twelve tips for using social media as a medical educator	We now live, learn, teach and practice medicine in the digital era. Social networking sites are used by at least half of all adults. Engagement with	Type of study: Perspective<br><br>Methodology:	Medicine	These practical tips help the newcomer to social media get started by identifying goals, establishing comfort, and connecting. Furthermore, users can	• The practical tips are: a) identify and reflect upon your digital identity and your own

Chretien, Katherine C.			social media can be personal, professional, or both, for health-related and educational purposes. Use is often public. Lapses in professionalism can have devastating consequences, but when used well social media can enhance the lives of and learning by health professionals and trainees, ultimately for public good. Both risks and opportunities abound for individuals who participate, and health professionals need tips to enhance use and avoid pitfalls in their use of social media and to uphold their professional values.	This article draws upon current evidence, policies, and the authors' experiences to present best practice tips for health professions educators, trainees, and students to build a framework for navigating the digital world in a way that maintains and promotes professionalism.		ultimately successfully contribute, engage, learn, and teach, and model professional behaviors while navigating social media.	goals; b) observe and establish comfort first. Think, then contribute. Lurk before you leap; c) apply existing social media guidelines, and develop individual guiding principles d) use social media to disseminate evidence-based health information, enhancing public health; e) engage, learn, teach, mentor: reflect on process; f) tap into the power of a community and advance your academic productivity by expanding your professional network
Knoll, M. A. and R. Jagsi	2019	Cumulative Micro-Mentorship: How Social Media Is Facilitating the Advancement of Female Physicians	The eruption of physician engagement on social media platforms has created unprecedented opportunities for female physicians to connect with each other, transcending common networking barriers including location, specialty, institution, and age. This connectivity facilitates an exchange of ideas and the opportunity to address unsolved issues for women physicians in what we suggest should be recognized as a highly powerful form of "cumulative micro-mentorship."	N.A.	Female physicians	Type of Study: Perspective<br>Methodology: N.A.	• Social media offers an unprecedented opportunity for female physicians in particular to both give and accept support in a repetitive and continual fashion but also to influence the overall systems within which they work to promote broader changes.<br>• Even with the limited scope of any individual interaction, the accumulation of feedback the system facilitates can have a large impact on the

							medical profession as a whole.
Laurence, C. E., Jones, J. R., Stone, S. N., Moses-Hampton, M., Yates, S. J., Khalil, M. E., … Strowd, R. E.	2020	Feasibility and Impact of a Student-Led, Semi-Structured, Near-Peer Student Guides Program on Navigating Through Medical School	Barriers to cultivating peer mentor relationships included limited interclass communication, difficulty identifying interested peers, and time burden on senior mentors. In response, the Navigating Medical School (NMS) Student Guides Program was designed, which pairs first year medical student (MS1) guidees with self-selected near-peer fourth year medical student (MS4) guides. Guides receive formal training in leadership, career development, and social support. Here, we report on the program's initial feasibility, usability, and impact, and characterize barriers to implementation and lessons learned.	Type of study: Convergent parallel mixed methods study<br><br>Methodology:<br>The feasibility, usability, professional and social impact, and barriers to implementation of a 16-week semi-structured, near-peer, student guides program involving 39 first year medical students (MS1s) and 41 fourth year medical students (MS4s) was assessed. Student enrollment was quantified, guide-guidee meetings tracked, and > 2 meetings defined as feasible. Meeting topics, impact on student advising, and barriers to sustainability were contextualized qualitatively.	First year and fourth year medical students	N.A.	• The Student Guides Program successfully engaged MS1s and MS4s; however, scheduling issues ultimately complicated guides' and guidees' ability to meet the requisite two times during the pilot period.<br>• Outcomes from our pilot reinforce the advantages of near-peer guides for junior and senior students.<br>• Our program adopted a semi-structured format to ensure that meeting content was responsive to the needs and interests of students while still fulfilling the overarching program goals to reinforce academic and career preparation and social support.<br>• The Student Guides Program was also uniquely integrated within the larger NMS framework, allowing guidees to access information and hear their questions reflected in a broader forum as well.
Levine et al	2017	Training the Workforce: Description of a Longitudinal	The increase in demand for palliative care (PC) ser- vices, with the coinciding acute shortage of hospice and palliative medicine (HPM)	Interventional: Thirty nurse and physician fellows representing 22 health systems	Mentees were fellows interest in PC. Mentors were seasoned	Enduring, interdisciplinary relationships were built at all levels across health care organisations. Fellows made significant increases in	• This interdisciplinary PC training model addressed local workforce issues by

		Interdisciplinary Education and Mentoring Program in Palliative Care	physicians,1 has led to concerns about clinician burnout and threats to long-term sustainability of programs.2 Given this, emphasis is being made to increase training in basic PC skills for primary care, subspecialty, and other interdisciplinary clinicians.3,4 Many existing programs offer valuable educational opportunities, some with the addition of brief mentoring support.5e7 Mentor- ing in medicine is an established means of profes- sional development, primarily in the academic setting, where success is linked to faculty develop- ment, research productivity, well-being, and job satisfaction.8e13 Yet, little is known about the value of mentoring in PC education and program develop- ment (PD), particularly in community-based settings. In 2011, the Chicago Regional Palliative Medicine Phy- sicians' Collective was formed to identify local gaps in PC services and establish a collaborative network among interdisciplinary providers. It comprises local experts representing nonprofit hospices, academic medical centers, a safety net hospital, and community-based hospitals. With support from a local nonprofit organisation, the Collective established the Coleman Palliative Medicine Training Program (CPMTP).<br>The CPMTP is a two-year training program for phy- sicians and nurses at Chicago area health systems seeking to increase access to PC services. Program goals are to 1) identify and address gaps to high- quality care, 2) train interdisciplinary clinicians in primary PC, 3) provide structured longitudinal mentorship to new and developing programs, and 4) build an enduring supportive regional network	across the Chicago region participated in a two-year PC training program. The curriculum was delivered through multiple conferences, self-directed e-learning, and individualized mentoring by expert local faculty (mentors). Fellows shadowed mentors' clinical practices and received guidance on designing, implementing, and evaluating a practice improvement project to address gaps in PC at their institutions.	clinicians and educators in paediatric and adult PC	knowledge and self-reported confidence in adult and pediatric PC and program development skills and frequency performing these skills. Fellows and mentors reported high satisfaction with the educational program.	increasing the number of clinicians capable of providing PC. Unique features include individualized longitudinal mentoring, interdisciplinary education, on-site project implementation, and local network building. Future research will address the impact of the addition of social work and chaplain trainees to the program<br>• The predominant limitations to this model are the time requirement of all parties and the necessary financial support. In addition, a comprehensive pro- gram evaluation was not conducted to ascertain which program components were most impactful. Data collection on the impact of mentorship and PIPs over time is ongoing.<br>• The following should be considered when creating a similar wide-ranging educational program: 1. Future work should evaluate the use of structured men- toring programs to assist with local PC team building and sustainability. 2.

			of PC clinicians. This article describes the development, implementation, and evaluation of the CPMTP from 2013 to 2015. Further evaluation of the program impact on patient outcomes and growth of PC teams is ongoing and will be published separately.				Institutional commitment goes beyond a letter of support. 3. Pediatrics faces unique challenges. 4. Health care climates are constantly in flux. 5. In this field, we need to take care of each other. 6. Cross-organisational collaboration is central to building a wide-ranging educational program.
Lin et al	2018	Mentoring in Nursing: An Integrative Review of Commentaries, Editorials, and Perspectives Papers	Mentoring in nursing is poorly understood and delineated and lacks a universal definition or a consistent approach	Built on a constructivist approach and use of the Grounded theory draws on accounts of nurse mentoring in editorials and perspective, reflective, narrative, and opinion pieces to create a narrative	Nursing	Thematic analysis of the 35 included articles revealed 5 themes. These themes include common features of mentoring among featured descriptions and definitions, mentoring relationships, mentor-related aspects, host organisation–related aspects, and mentee-related aspects.	• Mentoring is goal-specific, context-sensitive, and mentee-, mentor-, and organisation dependent suggests that a standardized mentoring framework can be used in nursing.<br>• Mentoring is also dependent on the quality of mentoring relationships, highlighting 5 considerations.<br>• One, matching mentors to mentees with similar motivations, interests, goals, and complementary personalities is pivotal to building personal ties.<br>• Two, awareness of the desired and undesired characteristics of mentee and mentor influences design mentor training and

							prepares mentees for mentoring.<br>• Three, it is important that there is willingness and agreement by mentors and mentees to work together. This highlights the importance of pre-mentoring meetings and interactions with agreement on goals, timelines, code of conduct, roles and responsibilities, and modes of communication.<br>• Four, the importance of a nurturing, trusting, and conducive mentoring environment that will foster frank exchange of ideas and open communications<br>• Five, organisational support for the mentoring process is important.<br>• The role of the host organisation lies beyond the provision of financial and administrative support for the mentoring program, mentor training, and mentor matching.<br>• Accounts of mentoring within editorials, opinion and perspective pieces, and commentaries echo the findings of more

							conventional reviews of mentoring. This data extends understanding of mentoring relationships and the influence of organisational factors and commonalities in the practice of mentoring, which are critical to building mentoring programs and neglected in the extant literature thus far.
Luckhaupt, S. E., Chin, M. H., Mangione, C. M., Phillips, R. S., Bell, D., Leonard, A. C., & Tsevat, J.	2005	Mentorship in academic general internal medicine. Results of a survey of mentors	To describe current trends in mentorship in general Internal Medicine (GIM).	Type of study: Cross-sectional survey<br><br>Methodology:<br>The authors conducted a national cross-sectional web-based survey of GIM mentors, GIM fellowship directors, and GIM National Institutes of Health K24 grant awardees to capture their experiences with mentoring, including compensation for mentorship, multiple mentees, comentorship, and long-distance mentorship. The authors compared experiences by mentorship funding status, faculty type, academic rank, and sex.	General Internal Medicine	Data was collected from 111 mentors (77% male, 54% full professors, and 68% clinician-investigators). Fifty-two (47%) received funding for mentorship. Mentors supervised a median (25th percentile, 75th percentile) of 5 (3, 8) mentees each, and would be willing to supervise a maximum of 6 (4, 10) mentees at once. Compared with mentors without funding, mentors with funding had more current mentees (mean of 8.3 vs 5.1, respectively; P<.001). Full professors had more current mentees than associate or assistant professors (8.0 vs 5.9 vs 2.4, respectively; P=.005). Ninety-four (85%) mentors had experience comentoring, and two-thirds of mentors had experience mentoring from a distance. Although most mentors found long-distance mentoring to be less demanding, most also said it is less effective for the mentee and is personally less fulfilling.	• Mentors in GIM appear to be close to their mentorship capacity, and the majority lack funding for mentorship.<br>• Comentoring and long-distance mentoring are common.
Masters, Ken; Ellaway, Rachel H; Topps, David;	2016	Mobile technologies in medical education:	This Guide builds upon the previous AMEE Guide to e-Learning in medical education by providing	Type of study: AMEE Guide	N.A.	N.A.	• Mobile technologies have developed rapidly, and medical

Archibald, Douglas, and Hogue, Rebecca J		AMEE Guide No. 105	medical teachers with conceptual frameworks and practical examples of using mobile technologies in medical education. The goal is to help medical teachers to use these concepts and technologies at all levels of medical education to improve the education of medical and healthcare personnel, and ultimately contribute to improved patient healthcare.	Methodology: N.A.			teachers can harness their power to improve medical teaching. To do so, medical teachers need knowledge of some socio-theoretical concepts, pedagogy, availability of appropriate applications and some basic technical expertise. They will also need to have the courage to implement changes to utilize these technologies. This Guide equips medical teachers with the tools to begin that process.
Nadir, Rans; Bashir, Khayam; Nasir, Mustafa; Abdulsalam, and Khan, Hassan Ali	2018	Social Networking Sites (SNSs) in medical education- A student's perspective	Describe the use of social networking sites in medical education	Type of study: Perspective<br><br>Methodology: N.A.	4th-year Medical Student	Given our own experiences in using various forms of social media for our education and the observed ubiquity of SNSs in our generation, we believe there is an important part for them to play in the future.	• The students could be provided with a framework to share resources such as a ready-made Facebook 'group' or made aware of specific features, notably the 'Group Video Call'.
Nicolai, Leo; Schmidbauer, Moritz; Gradel, Maximilian; Ferch, Sabine; Antón, Sofía; Hoppe, Boj; Pander, Tanja; von der Borch, Philip; Pinilla, Severin, and Fischer, Martin	2017	Facebook Groups as a Powerful and Dynamic Tool in Medical Education: Mixed-Method Study	A clear picture on educational usage of Facebook groups has yet to emerge to assess to what extent medical faculties can take educational advantage of these networks. In particular, there is a lack of data on user motivation, user typologies, subjective benefits, and limitations, as well as patterns of posting behaviour of students necessary for further analysis and integration into existing (learning) theories.	Type of study: Mixed-Methods Study<br><br>Methodology:<br>For characterization of medical students involved in Facebook groups, we conducted focus groups among Facebook users and structured interviews of specific student groups (social media drivers and students not using Facebook here called new	Preclinical Medical Students in Ludwig-Maximilians-University	All focus group participants (n=21, 12 female, and 9 male students) were registered on Facebook and were using semester-spanning groups. Motivation for setting up a Facebook account was mainly socializing and staying in touch with (international) friends and acquaintances. Nevertheless, 2 students registered solely for being able to join the aforementioned student groups.	• All focus group participants were registered on Facebook and were using semester-spanning groups. Motivation for setting up a Facebook account was mainly socializing and staying in touch with (international) friends and acquaintances. Facebook was used for educational as well as private purposes on a

				minorities). For the evaluation of posting behaviour and for identification of covered topics, we combined qualitative and quantitative methods to analyse posts in two semester-spanning Facebook groups. Structured interviews focused on reasons for Facebook abstinence, and alternatives for information gathering were conducted (n=6).			daily basis. When asked about their behaviour and motivation for extensive posting in the respective groups, we identified two distinct subtypes: Some social media drivers we interviewed see themselves as service providers, answering questions and providing information as well as resources. Altruism was named as the main reason for this dedication. Further reasons were access to exclusive information as well as the feeling of an obligation to return a favor. The second subtype comprised students that perform below average and use Facebook groups to get support. Contrary to the first subtype that contributes in sharing information, this subgroup seems to post more questions.
Olaussen, Alexander; Reddy, Priya; Irvine, Susan, and Williams, Brett	2016	Peer-assisted learning- Time for nomenclature clarification	The vogue of social media has changed interpersonal communication as well as learning and teaching opportunities in medical education. The most popular social media tool is Facebook. Its features provide potentially useful support for the education of medical students but it also means that some new challenges will have to be faced.	Type of study: Scoping review<br><br>Methodology<br>The authors searched six databases. Inclusion criteria were defined and the authors independently reviewed the search results. The key	Unspecific	16 articles met all inclusion criteria. 45-96% of health care professionals in all phases of their medical education have a Facebook profile. Most studies focused on Facebook and digital professionalism. Unprofessional behavior and privacy violations occurred in 0.02% to 16%. In terms of learning and teaching environment, Facebook is well accepted by medical students. It is used to prepare for	• We suggest herein a simple pragmatic terminology to overcome ambiguous terminology. Academically, clear terms will allow effective and efficient research, ensuring furthering of the

				information of the articles included was methodically abstracted and coded, synthesized and discussed in the categories study design, study participants'phase of medical education and study content.		exams, share online material, discuss clinical cases, organize face-to-face sessions and exchange information on clerkships. A few educational materials to teach Facebook professionalism were positively evaluated. There seems to be no conclusive evidence as to whether medical students benefit from Facebook as a learning environment on higher competence levels.	educational philosophy. To broaden the platform of research around PAL and to allow easy integration across institutions, consistent terms and definitions are necessary. We urge consistent use of the PAL terms based on the suggested groupings offered in this paper. Expansion of the MeSH (Medical Subject Headings) terms is necessary. It may be anticipated that new terminology introduction may be inconvenient at first, and it is unlikely that a consensus will be reached quickly; however, we believe the long-term benefits uniform terminology has on research and education outweigh this hindrance.
O'Mahony, S. Baron, A. Ansari, A. Deamant, C. Nelson-Becker, H. Fitchett, G. Levine, S.	2020	Expanding the Interdisciplinary Palliative Medicine Workforce: A Longitudinal Education and Mentoring Program for Practicing Clinicians	The disparity between gaps in workforce and availability of palliative care (PC) services is an increasing issue in health care. To meet the demand, team-based PC requires additional educational training for all clinicians caring for persons with serious illness. This paper aims to describe the educational methodology and evaluation of an existing regional interdisciplinary PC training program that was expanded to include chaplain and social worker trainees.	Type of study: Interventional with Qualitative Feedback<br><br>Methodology: From 2015 to 2017, 26 social workers, chaplains, physicians, nurses, and advanced practice providers representing 22 health systems completed a two-year training program. The curriculum comprises biannual	Physicians, nurses, advance practice providers, social workers, chaplains	Among 12 skills common to all disciplines, trainees reported significant increases in confidence across all 12 skills and significant increases in frequency of performing 11 of 12 skills. Qualitative evaluation identified a myriad of program strengths and challenges regarding the educational format, mentoring, and networking across disciplines.	• Teaching PC and program development knowledge and skills to an interdisciplinary regional cohort of practicing clinicians yielded improvements in clinical skills, implementation of practice change projects, and a sense of belonging to a

				interdisciplinary conferences, individualized mentoring and clinical shadowing, self-directed e-learning, and profession-focused seminar series for social workers and chaplains. Site-specific practice improvement projects were developed to address gaps in PC at participating sites. PC and program development skills were self-assessed before and after training.			supportive professional network.
Pander, Tanja; Pinilla, Severin; Dimitriadis, Konstantinos, and Fischer, Martin R.	2014	The use of Facebook in medical education – A literature review	The vogue of social media has changed interpersonal communication as well as learning and teaching opportunities in medical education. The most popular social media tool is Facebook. Its features provide potentially useful support for the education of medical students but it also means that some new challenges will have to be faced.	Type of study: Systematic Review<br><br>Methodology:<br>The authors searched six databases. Inclusion criteria were defined and the authors independently reviewed the search results. The key information of the articles included was methodically abstracted and coded, synthesized and discussed in the categories study design, study participants' phase of medical education and study content.	Undergraduate medical education, graduate medical education and continuing medical education	16 articles met all inclusion criteria. 45-96% of health care professionals in all phases of their medical education have a Facebook profile. Most studies focused on Facebook and digital professionalism. Unprofessional behavior and privacy violations occurred in 0.02% to 16%.	• Facebook influences a myriad of aspects of health care professionals, particularly at undergraduate and graduate level in medical education. Despite an increasing number of interventions, there is a lack of conclusive evidence in terms of its educational effectiveness. Furthermore, we suggest that digital professionalism be integrated in established and emerging competency-based catalogues.
Patel, Pradip D.; Roberts, John L.; Miller, Karen; Hughes; Ziegler,	2012	The Responsible Use of Online Social Networking Who Should	As medical students become more active in online social networking (OSN), there are increasing concerns	Type of study: Quantitative study<br><br>Methodology:	3rd-year Medical Students, 1st-year Residents, and Clinical Faculty	We found residents to be more like students in usage patterns of personal electronic media and in their choice of the mentoring techniques that should	• Facebook influences a myriad of aspects of health care

Craig, and Ostapchuk, Michael		Mentor Medical Students	regarding violations of patient privacy and a lack of professionalism.	We surveyed faculty (N = 16), 1st-year residents (N = 120), and 3rd-year medical students (N = 130) to compare attitudes about OSN and the online usage patterns.		be used. Residents say they were not prepared to mentor students without additional guidance but were more confident than faculty members that they had the knowledge to do so.	professionals, particularly at undergraduate and graduate level in medical education. Despite an increasing number of interventions, there is a lack of conclusive evidence in terms of its educational effectiveness. Furthermore, we suggest that digital professionalism be integrated in established and emerging competency-based catalogues.
Patricia Reid Ponte, RN, DNSc, FAAN, NEA-BCa,b, Laura L. Hayman, PhD, RN, FAAN, FAHAc Donna L. Berry, RN, PhD, AOCN, FAANd,e Mary E. Cooley, APRN, BC, PhD, RN, FAANc,d	2015	A new model for postdoctoral training: The Nursing Postdoctoral Program in Cancer and Health Disparities	The University of Massachusetts Boston and Dana-Farber/Harvard Cancer Center joined forces in 2009 to create a Postdoctoral Nursing Research Fellow- ship in Cancer and Health Disparities. In combining the resources of a large university and a research-intensive service institution, the postdoctoral program provides a new model for preparing nurse scientists to conduct independent research that advances nursing knowledge and interdisciplinary understanding of complex health issues. The multifaceted program consists of educational programming, research training, and career planning components. Additionally, each fellow is assigned a nurse scientist mentor and interdisciplinary co-mentor. The mentors support the fellows with scholarly activities and research training and help the fellows craft individualized career plans, including proposals for post fellowship career development research.	Interventional: In this article, the postdoctoral program leaders describe the program structure, strategies used to recruit minority and nonminority candidates, and data describing program outcomes and share lessons learned and recommendations for organizations that may be interested in establishing similar postdoctoral fellowships at their institutions.	Nursing	Outcomes achieved by the Postdoctoral Nursing Research Fellowship, including the depth and diversity of topics researched by trainees, the number of accepted manuscripts and presentations, the success experienced by some fellows in securing grant funding, and favourable ratings of the fellowship experience, suggest the program was both well received by the majority of participants and effective in helping them acquire the skills needed for an independent research career.	• A number of factors contributed to the program's success. Perhaps most important is the long-standing partnership between UMB and DF/HCC and the commitment of the institutions' nurse leaders to advancing and expanding educational opportunities for minority and nonminority nurses. The resources made available by the two institutions assure that nurse fellows have ample support for developing new skills and knowledge as well as opportunities for interacting and

							collaborating with experienced nurse and interdisciplinary scientists with complementary research interests.<br>• Also key to the program's success is the availability of experienced and highly skilled nurse scientists and interdisciplinary researchers willing to serve as men-tors. As indicated by the evaluation survey, the mentors and mentoring component were key factors influencing nurses to apply to the program as well as a major source of satisfaction. Recognizing the mentor-ing component's importance, program leaders have taken steps to further strengthen mentoring relation- ships by assuring junior mentors receive individual support and guidance in identifying and meeting mentee needs.<br>• One factor that may contribute to the program's long-term success is the availability of the non-traditional option.<br>• Another lesson learned involves the

							assignment of mentors. Matching fellows with mentors who share their goals and research interests not only assures a more satisfying experience for fellows and mentors but also results in more joint/collaborative publications and presentations in peer-reviewed nursing and interdisciplinary forums and enhances the likelihood of successful outcomes with grant submissions<br>• In this era of team science, it is helpful to supplement the fellow-mentor relationship with an inter-professional mentoring team composed of active investigators from disciplines and professional fields that are relevant to the fellow's short- and long-term research goals.<br>• The makeup of the leadership team that is charged with overseeing program development and operations is yet another area that merits close attention by organizations seeking to establish a

							postdoctoral pro- gram in nursing research
Periyakoil	2007	Declaration of Interdependence: The Need for Mosaic Mentoring in Palliative Care.	This editorial highlights the role of mentoring in PM which includes sponsoring, facilitator, tutor, role modelling, coaching and advising. An effective mentoring mosaic is an intra- institutional and inter-institutional matrix of senior colleagues, teachers, peers, as well as junior colleagues and students who provide ongoing multifaceted input and counseling and also facilitate access to a variety of resources in a mutually beneficial way. This type of a multilayered, multifaceted mentoring mosaic is especially critical for the creation, growth, and sustenance of the next generation of leaders	Type of study: Narrative	Palliative care mentor	N.A.	• IPM is competency based relationship which involves a two way information and resource sharing system using the concepts of reflexivity and teamwork palliative care junior faculty have complex needs that are well beyond the scope of any one individual mentor
Pinilla, S., Nicolai, L., Gradel, M., Pander, T., Fischer, M. R., von der Borch, P., Dimitriadis, K.	2015	Undergraduate Medical Students Using Facebook as a Peer-Mentoring Platform: A Mixed-Methods Study	To describe and explore the Facebook use of undergraduate medical students during their first 2 years at a German medical school. The data should help medical educators to effectively integrate social media in formal mentoring programs for medical	Type of study:<br>Mixed methods study<br><br>Methodology:<br>The authors developed a coding scheme for peer mentoring and conducted a mixed-methods study in order to explore Facebook groups of undergraduate medical students from a peer-mentoring perspective	Medical students	All major peer-mentoring categories were identified in Facebook groups of medical students. The relevance of these Facebook groups was confirmed through triangulation with focus groups and descriptive statistics. Medical students made extensive use of Facebook and wrote a total of 11,853 posts and comments in the respective Facebook groups (n=2362 total group members). Posting peaks were identified at the beginning of semesters and before exam periods, reflecting the formal curriculum milestones	• Peer mentoring is present in Facebook groups formed by undergraduate medical students who extensively use these groups to seek advice from peers on study-related issues and, in particular exam preparation.<br>• These groups also seem to be effective in supporting responsive and large-scale peer-mentoring structures; formal mentoring programs might benefit from integrating social media into their activity portfolio.
Pinilla, S., Pander, T., von der Borch, P., Fischer, M. R., & Dimitriadis, K	2015	5 years of experience with a large-scale mentoring program	Describe the features of the MeCuM-Mentor	Type of study:<br>Descriptive<br><br>Methodology:	Two tiered:<br><br>Preclinical –	N.A.	• Two tiered program is well accepted by students and faculty.

		for medical students		A needs analysis among medical students in all semesters was conducted. There is an overall demand for personal and professional development as well as career planning. A two tiered mentoring program consisting of a 1:1 mentoring program for clinical students and a peer mentoring program with a focus on preclinical students. The students' and mentors' participation at the program is voluntary. Majority of the matching was done online where student and physicians complete online profiles consisting of 13 items with regards to their professional background and career interests as well as to their work-life priority. A 6-level Likert scale and a free text section was used. A computer-based matching score using a weighted correlation algorithm presents student with the 10 mentors who are most likely to suit their profiles.	Mentees are preclinical students and mentors are junior mentors from all semesters.<br><br>Clinical – Mentees are clinical students and mentors are physicians with different backgrounds and from different disciplines and specialities.		The online based matching seems to be effective for large scale mentoring programs.<br>• Voluntary participation is important, as intrinsic motivation is condition as well as foundation of a programs' success. Peer -mentoring is also very receptive. No consensus on what kind of matching process would be ideal for a mentoring programme in medical school.
Pollack, T. M. et, al.	2020	Building HIV healthcare worker capacity through telehealth in Vietnam	Health worker training and mentoring has been an integral component of the scale-up of antiretroviral	Type of study: Interventional Methodology: To monitor implementation and	Healthcare workers	More than 85% of respond-ents agreed that access to the telehealth programme had improved the quality of care they provided to their patients. Eighty-five per cent reported that the	• Four key issues have been identified as necessary to promote sustainability and country ownership of

			therapy around the world. Scalable and sustainable models of health worker capacity building are needed to support national HIV programmes in low and middle-income countries. Telehealth is a novel approach that uses live online videoconferencing technology to connect remote health workers with experts from specialty centres for the purpose of training, mentoring and support of programme implementation. Lessons learnt from Vietnam's experience can provide national programmes and training institutions with approaches to designing, implementing and sustaining HIV telehealth programmes in their settings.	track the scale-up of the programme, we routinely collected data from each hub on the number of sessions, number of participating sites per session and number of participants per session. In addition, to assess the effectiveness of the programme and participant satisfaction, we conducted an online survey in April 2018.		sessions were easy to access and the majority reported session technical quality (internet access, sound and picture) as good or very good. Nearly 90% reported that they would like to continue to participate in future telehealth sessions.<br>Survey respondents also reported a change in how they access CME following participation in the telehealth programme. The proportion reporting the need to travel outside one's own province for training decreased from 25.5% to 17.5% and live online training participation increased from 26.4% to 40.1%. Live online training<br>became the most common reported way respondents accessed CME. Additionally, 53.8% reported being willing to pay for CME credits obtained through telehealth.	PEPFAR initiatives: aligning resources with national government health strategies and plans, pursuing a range of financing strategies, establishing evidence-based service delivery models adaptable to advancements in HIV prevention and treatment and ensuring adequate models of healthcare provider training and capacity building led by national institutions.<br>• Our experience demonstrates that a live videoconferencing telehealth approach can help establish an HIV TA platform led by national institutions.<br>• HIV telehealth is a scalable, sustainable and effective approach to building HIV health worker capacity.
Polley Clare	2020	A novel approach to medical mentoring	Describe the JRMO mentoring programme	Type of study: Descriptive<br><br>Methodology:<br>To determine the best model for a JRMO mentoring programme they conducted a literature review of mentoring in medicine, and approaches from non-medical literature, in conjunction with an online survey of past JRMOs to understand	Junior resident medical officers, in PGY 2–4, are invited to participate in a self-selected dyad mentoring programme. The mentors volunteer come from the registrar and fellows from PGY3 to 6 cohorts Thirty JRMO mentees and 36	The self-selected dyad model, using mentor videos and online resources housed on the RCH learning management system, was designed to maximise the flexibility, sustainability and accessibility of the programme. A brokering and support system for the mentors and the mentees is part of the model.	• Most common approach is face to face followed by emails.<br>• Mentoring profiles was the most useful tool, followed by "the first meeting" document. The self-selection model was key for the engagement of mentees. Fundamental to the success of our programme are the mentors' video

				their mentoring needs. They established that JRMOs preferred a model using a near-peer mentor in a dyad relationship. They allowed mentees to choose their mentor based on their personal priorities, including gender, subspecialty or particular skills, such as research, as the literature indicates that matching for these attributes adds to the strength of the relationship.<br>End of year survey was done where 56/60 people responded.	mentors opted in to the programme, resulting in 30 matched pairs.		profiles. These profiles provided 'stories', offering an insight into mentors' lives, activities and interests.<br>• Fundamental to the success of our programme are the mentors' video profiles. These profiles provided 'stories', offering an insight into mentors' lives, activities and interests.
Martin, Priya; Kumar, Saravana, and Lizarondo, Lucylynn	2017	Effective use of technology in clinical supervision	Clinical supervision is integral to continuing professional development of health professionals. With advances in technology, clinical supervision too can be undertaken using mediums such as videoconference, email and teleconference. While telesupervision could be useful in any context, its value is amplified for health professionals working in rural and remote areas where access to supervisors within the local work environment is often diminished.	Type of study: Literature review<br><br>Methodology:<br>This article outlines ten evidence-informed, practical tips stemming from a review of the literature that will enable health care stakeholders to use technology effectively and efficiently while undertaking clinical supervision.	Clinical, continuing professional development healthcare professionals	N.A.	• Telesupervision offers an opportunity to overcome the tyrannies of distance, access and time. However, improved access to technology and connectivity does not necessarily equate to quality telesupervision. Based on contemporary research evidence and real-world experience from the coal face, this article provides ten practical tips that will enable health care stakeholders to use technology effectively and efficiently through all the stages of telesupervision namely

							pre-session, in-session and post-session.
Quentin R. Youmans, Jennifer A. Adrissi, Adesuwa Akhetuamhen, Khalilah L. Gates, Aashish K. Didwania, Diane B. Wayne, & Linda I. Suleiman	2020	The STRIVE Initiative: A Resident-Led Mentorship Framework for Underrepresented Minority Medical Students	Underrepresented minority (URM) trainees face unique challenges in academic medicine. Near-peer mentorship is an under-described method to support URM trainees.	Type of study: Quantitative study<br><br>Methodology:<br>All URM residents were invited to participate in the STRIVE mentorship program consisting of 3 pillars of programming: medical school curriculum review sessions, panel discussions, and social events for medical students. The program was evaluated through participation rates and a 7-item survey delivered in May 2019 after 3 years of implementation.	Residents (as mentors) and Medical Students (as mentees)	The STRIVE initiative conducted 25 events. Thirty-five of 151 eligible (23%) URM residents participated as mentors for an average of 50 of 110 eligible (45%) URM medical students annually. Resident mentors participated for an average of 3 to 4 hours each year. Twenty of 32 eligible resident mentors (63%) completed the survey. Ninety-five percent (19 of 20) of survey respondents agreed that STRIVE made them a better mentor; 90% (18 of 20) reported that they would have appreciated an equivalent program during their medical school training; and 75% (15 of 20) agreed that the program helped them address the challenges of underrepresentation in medicine.	• Student to Resident Institutional Vehicle for Excellence (STRIVE) near-peer mentoring program was created in a large urban medical school and associated residency programs. All URM residents were invited to participate in the STRIVE mentorship program consisting of 3 pillars of programming: medical school curriculum review sessions, panel discussions, and social events for medical students.<br>• The STRIVE programme made the residents better mentors. They reported that they would have appreciated an equivalent program during their medical school training and agreed that the program helped them address the challenges of underrepresentation in medicine.<br>• Over a 3-year period, STRIVE required a modest amount of resident time and was valued by the URM residents and medical students who

							participated in the program.
Raiman, Lewis; Antbring, Richard, and Mahmood, Asad	2017	WhatsApp messenger as a tool to supplement medical education for medical students on clinical attachment	Instant messaging applications have the potential to improve and facilitate communication between hospital doctors and students, hence generating and improving learning opportunities. This study aims to demonstrate the feasibility and acceptability of instant messaging communication to supplement medical education for medical students whilst on clinical attachment.	Type of study: Interventional WhatsApp Group for PBL in Clinical Students/Thematic Analysis<br><br>Methodology:<br>A total of 6 WhatsApp Messenger (WhatsApp Inc.) groups were created for medical students on clinical attachment. These were used to provide communication within Problem Based Learning (PBL) groups for a duration of 8 weeks. The frequency and type of communication were recorded. Students' opinions were evaluated through a structured interview process at the end of the study period. A thematic analysis was performed on the content of the instant messaging groups and on the results of the structured interviews.	3rd year medical students attending medical and surgical clinical rotations and Problem Based Learning (PBL)	All of the participants were active in their respective messaging groups (19 students and 6 tutors). A total of 582 messages, 22 images and 19 webpage links were sent. Thematic analysis on content of the instant messaging groups identified the following themes: organisational, educational and social. Thematic analysis on the content of interviews identified themes such as the ease of use of instant messaging, benefit of instant messaging to foster understanding and learning, and the ability to access recorded discussions.	• The contents of the WhatsApp group can be divided into organizational, educational and social aspects. Findings gathered from the feedback interview includes the ease of use of instant messaging, benefit of instant messaging to foster understanding and learning, the sharing of resources electronically, the access of recorded discussions, the generation of learning opportunities outside PBL, the intrusiveness of instant messaging and the lack of face-to-face interaction.
Rastegar Kazerooni, A. Amini, M. Tabari, P. Moosavi, M.	2020	Peer mentoring for medical students during COVID-19 pandemic via a social media platform	Students find themselves coping with mental and emotional issues, including stress, anxiety, and fear, that may require significant psychological and physical effort. Therefore, it is important that medical schools not only care about students' mental health but also implement strategies to support their understanding of crisis management,	Type of Study: Interventional<br><br>Methodology:<br>A social media platform was created that employed the Near Peer Mentoring method by having senior medical	Medical Students	A valid and reliable questionnaire, based on our previous study, was used to measure the effect of this activity. 71% of junior medical students believed the platform had a significant impact on helping them adjust faster to these emergency conditions. The only barriers mentioned by the students was that some were not sure how to apply the	• Care and support have a critical role in promoting mental health in medical students. Building a peer mentoring group can help.<br>• Keeping in mind the concept of "Teaching

			self-mental care, and other principal measures in order to strengthen their coping skills and mental preparedness. In collaboration with students of Shiraz medical school, a social media platform was created that<br>employed the Near Peer Mentoring method by having senior medical students instruct junior medical<br>students in coping with the anxiety and stress brought by COVID-19 pandemic.	students instruct junior medical students in coping with the anxiety and stress brought by COVID-19 pandemic. These senior students had about 40 hours of training in teaching and learning methods, communication skills, and effective consulting techniques under the supervision of faculty during the preceding three years. 371 junior students joined this group. Students communicated with each other, exchanging thoughts and feelings about mental and psychological issues. Ten senior students, under the supervision of expert faculty, offered suitable solutions to psychological concerns and supported junior students in managing their emotions about this highly contagious disease. They recommended stress relaxation techniques, engaging in exercise, remaining in online contact with family and friends, time management during quarantine, etc. General discussion about the best learning strategies for online classes also took place.		information gained to their personal situation as the<br>continued to desire real and practical face-to-face consultation. Nonetheless, students generally<br>reported this activity was a unique experience that was beneficial for their professional growth.	is Learning Twice," we have learned that by taking on peer mentoring leadership responsibility, senior students can further the competencies they need to be competent physicians.

Rogers, K. M. Childers, D. J. Messler, J. Nolan, A. Nickel, W. K. Maynard, G. A.	2014	Glycemic control mentored implementation: creating a national network of shared information	The Society of Hospital Medicine's (SHM's) Glycemic Control Mentored Implementation (GCMI) program, which, like all MI programs, is conducted as an improvement collaborative, is intended to help hospitals improve inpatient glycemic control in diabetic and nondiabetic patients by educating and mentoring quality teams.	Type of study: Interventional with Qualitative Feedback<br><br>Methodology:<br>Hospital quality improvement (QI) teams applied for participation in GCMI from 2009 through 2012. Accepted sites were assigned either a hospitalist or endocrinologist mentor to work through the life cycle of a QI project. SHM's Implementation Guide, online resources, measurement strategies, Web-based Glycemic Control Data Center for Performance Tracking, webinars, interactive list-serve, and other tools help mentors guide these teams through the program. Mentors in GCMI bring expertise in both inpatient glycemic control and QI.	Hospital quality improvement teams	One hundred fourteen hospital QI teams were enrolled into the GCMI program in the course of 2.5 years. Of these 114 sites, 90 completed the program, with 63 of them uploading data to the Data Center. Feedback from the sites was consistently positive, with the listserve, Data Center, and mentorship reported as the top three most effective components of the program. Ninety-five percent of respondents stated that they would recommend participation in an SHM–mentored implementation program to a colleague. Participants reported improved leadership skills and increased institutional support for glycemic control.	• Hospital quality teams participating in the GCMI program gained support to overcome barriers, focus on improving glycemic control, network with peers and expert mentor physicians, collect and analyze data, and build quality leaders. The features and structure of this program can be used in other multisite QI goals and projects.
Rohrer, J. D. et. al	2014	Developing a neurology mentoring programme for trainees	The popularity of neurology compared with other medical specialities has declined in recent years. Changes in medical school curricula and in junior doctor training mean that many doctors have less exposure to neurology than previously. A national neurology mentoring programme was set up with the aim of supporting individuals who show an interest in neurology,	Type of study: Interventional<br><br>Methodology:<br>Mentors and mentees will be in contact via email with 1 or 2 face-to-face meetings per year. Mentors will be encouraged to invite mentees to local neurology rounds. A	Junior doctors interested in neurology	N.A.	N.A.

			enabling them to manage their career development in an effective manner.	standardised feedback form will be used to qualitatively report outcomes of the programme.			
Rosenzweig, M. Q. et al	2016	Primary care nurse practitioners providing survivorship cancer care	The Oncology Nurse Practitioner Web Education Resource (ONc-PoWER) is an online course developed specifically for nurse practitioners (NPs) in their first year of oncology practice paired with an onsite mentor (physician, nurse practitioner or physician assistant). The course consists of 5 interactive modules. The purpose of this study was to examine the NPs and mentors experience with the learning activities and to what degree the learning objectives were met.	Type of study: Interventional<br><br>Methodology:<br>Dyads of NPs and mentors completed the course over 4-6 months. There are items for course evaluation with Likert scaled responses of 1) did not meet objective 2) somewhat met objective 3) met objective 4) more than met objective 5) exceeded objective expectation.	Nurse practitioners	Thirty NPs new to practice and 22 oncology mentors have completed evaluations thus far. Responses overall are favorable.	• The ONcPoWER web enhanced oncology orientation program was favorably evaluated by nurse practitioners new to cancer care and their mentors.<br>• This method of electronic orientation could standardize the exposure of essential basic cancer care competencies at entry to oncology nurse practitioner practice and, with some edits to content, for community based primary care nurse practitioners caring for cancer survivors.
Rowe, Michael; Frantz, Jose, and Bozalek, Vivienne	2012	The role of blended learning in the clinical education of healthcare students: a systematic review	Developing practice knowledge in healthcare is a complex process that is difficult to teach. Clinical education exposes students to authentic learning situations, but students also need epistemological access to tacit knowledge and clinical reasoning skills in order to interpret clinical problems. Blended learning offers opportunities for the complexity of learning by integrating face-to-face and online interaction. However, little is known about its use in clinical education.	Type of study: Systematic review<br><br>Methodology:<br>Articles published between 2000 and 2010 were retrieved from online and print sources, and included multiple search methodologies. Search terms were derived following a preliminary review of relevant literature.	Healthcare students across a range of disciplines, including physiotherapy, medicine, nursing, social work, occupational therapy, pharmacy and paramedics	A total of 71 articles were retrieved and 57 were removed after two rounds of analysis. Further methodological appraisals excluded another seven, leaving seven for the review. All studies reviewed evaluated the use of a blended learning intervention in a clinical context, although each intervention was different. Three studies included a control group, and two were qualitative in nature. Blended learning was shown to help bridge the gap between theory and practice and to improve a range of selected clinical competencies among students.	• Clinical education is complex and requires a multifaceted approach to address the many needs of students. Blended learning may have some potential to enhance the development of a range of clinical competencies among healthcare students. There is a need for well-designed, well-described studies into the use of blended learning in clinical

							education, especially in developing countries.
Sandars, John; Homer, Matthew; Pell, Godfrey, and Croker, Tom	2008	Web 2.0 and social software: the medical student way of e-learning	A wide range of social software has become readily available to young people. There is increasing interest in the exciting possibilities of using social software for undergraduate medical education.	Type of study:<br>A structured self-administered questionnaire survey of 212 students.<br><br>Methodology:<br>A structured self-administered questionnaire survey of 212 students.	First Year Medical Students	Over 90 percent used instant messaging and social networking sites were highly used (70 percent). There was no significant difference between males and females. Blogs were read by about a fifth of students and a small number (8%) wrote their own blogs. A fifth of males stated that they were users of media sharing and contributed to wikis. Social bookmarking was rarely used by either sex.	• The use of social software amongst young people, including first year medical students, is very common. Social networking sites provide several combined features, including instant messaging and blogs, and could be used to develop personalised e-learning. Social software can be used for reflective diaries, portfolios, resources for lifelong learning and online discussions and group work. Social software should be integrated into existing curricula and Virtual Learning Environments (VLEs).
Saparova, Dinara; Williams, Jayne A; Inabnit, Christopher K, and Fiesta, Michelle	2013	Information Behavior Shift How and Why Medical Students Use Facebook	One prevalent aspect in previous studies is that use of social media tools was initiated by faculty, and student participation was often an additional course requirement. This could have negated the self-directed learning and sense of sharing freely. In this poster we demonstrate the use of the SNS group by students only. More specifically, we show how the SNS group that was created by medical students and for medical students enrolled in a PBL curriculum evolved overtime and what the manifestations of the associated with	Type of study:<br>Poster<br><br>Methodology:<br>Mixed methods approach to data collection and analysis allowed us to obtain a comprehensive overview of the SNS group usage. Qualitative and quantitative analyses of content in the described FB group were performed in	Undergraduate medical students	N.A.	• Patterns of the group utilization became the evidence of extending the classic principles of PBL in medical education into a digital learning environment, which, in its turn, results in gradual transformation of medical students' information behavior. By shifting from more traditional forms of communication and

			it trends in students' information behavior were.	order to identify thematic and chronological trends of the group use among medical students over a period of almost three academic years1. Focus group interviews with five students were also conducted to exemplify some of the reasons behind students' use of the SNS group.			information technology to using content available through social media, medical students incorporate SNS in their learning as an online environment that supplements their face-to-face interactions and facilitates acquisition and/or exchange of school-related information in easier, more efficient ways.
Schäfer, M., Pander, T., Pinilla, S., Fischer, M. R., von der Borch, P., & Dimitriadis, K.	2016	A prospective, randomised trial of different matching procedures for structured mentoring programmes in medical education	We set out to investigate and compare three methods of matchmaking in the setting of our structured mentoring programme. In a prospective, randomised trial we compared personal matching (PM) by an experienced expert to two different electronic data processing (EDP)-supported matching procedures: ''online algorithm'' (OA) versus ''online search'' (OS).	Type of study: Randomised trial<br><br>Methodology:<br>Personal matching(PM) was performed after structured interviews of prospective protégé's by founder of the mentoring programme.<br>The online algorithm, electronic data processing (EDP) provides students with a choice of 10 potential mentors based on comparison of online profiles. Online search lets students filter and search through all available mentor profiles. One hundred and ninety medical students were randomised into the three groups. One year later, they evaluated the endpoints	165 students found a mentor in the allocated matching procedure.<br>Two tiered:<br><br>Preclinical – Mentees are preclinical students and mentors are junior mentors from all semesters.<br><br>Clinical – Mentees are clinical students and mentors are physicians with different backgrounds and from different disciplines and specialities.	One hundred sixty-five out of the 190 study participants found a mentor. With regards to endpoint one we found an advantage of PM compared to both EDP-supported matching procedures. There was no significant difference between OA and OS. Concerning endpoint two the differences between the investigated matching procedures were not significant.	• PM is superior as to the number of mentoring relationships formed per participating student compared to Electronic data processing (EDP)-supported methods. In our data, there was no significant difference in the level of satisfaction.<br>Considering the high investments associated with PM of mentors and protégé's, EDP-supported matching procedures seem a viable compromise between effectiveness and efficiency and especially for large-scale structured mentoring programmes in medical education.

				1. ‘‘establishment of a mentoring relationship’’<br>2. ‘‘satisfaction with the mentoring relationship’’ using Munich-Evaluation-of-Mentoring-Questionnaire (MEMeQ).<br>All students were distributed equally to the three matching procedures by an automated randomization algorithm. As students could be excluded after randomization, the three groups did not remain exactly equal in number. We informed our mentors about the particular matching procedure of their proteˊgeˊs to ensure equal conditions for all groups.			
Schichtel, Markus	2009	A conceptual description of potential scenarios of e-mentoring in GP specialist training	The usefulness of face-to-face mentoring has been recognised in general practice for some time. E-learning is increasingly seen as an innovative way of supporting GPs in their continuing professional development. The fusion of e-learning and mentoring into e-mentoring is explored by a review of the literature an three potential GP specialist training scenarios are dicussed. E-mentoring is effective, enhances face-to-face interaction with colleagues and should be driven by learning needs and educational principles	Type of study: Perspective<br><br>Methodology: N.A.	Postgraduate general practice trainee	N.A.	• The fusion of e-learning and mentoring into e-mentoring is explored by a review of the literature. Three potential GP specialist training scenarios are discussed: a) e-mentoring for general practice specialist registrars; b) e-mentoring for educational supervisors and GP trainers; c) e-mentoring for clinical supervisors. E-mentoring is effective,

							enhances face-to-face interaction with colleagues and should be driven by learning needs and educational principles. A consultation with stakeholders through questionnaires and interviews to analyse current professional training needs in e-mentoring is strongly suggested. Pilot studies should evaluate the usefulness, usability and organisational acceptability of e-mentoring programmes within general practice specialist training.
Schichtel, M	2010	Core-competence skills in e-mentoring for medical educators: a conceptual exploration	The primary aim was to review and synthesise existing evidence in educational science that addressed the question: ‘‘what are the required core-competences in e-mentoring for medical educators?’’	Type of study: Systematic Review<br><br>Methodology:<br>A systematic literature review based on the Campbell Collaboration (2000) guidelines was seen as a suitable methodology to explore an analytical concept that explained the key issues in e-mentoring core-competences for medical educators.	Medical educators	A total of 4344 citations from the literature search were identified. Altogether 44 items were finally selected for inclusion in the study. The extant quality of published research was generally weak. The weight of the best available evidence suggested that seven specific e-mentoring core-competence domains were seen as significant.	• E-mentoring and e-learning skills training should occur during medical training.<br>• E-mentoring should become part of the training of the health care professionals<br>• Preferably collaborative development groups should be formed at local, regional and national level to design, initiate, execute and evaluate e-mentoring programmes.<br>• E-mentoring roles within virtual communities such as blogs, social networking sites or VLEs should become part of supporting

							health care professionals and medical educators especially during times of professional or organisational change.
Scott et. Al.	2019	The Rising Physicians Program: A Novel Approach for Mentoring Medical Students	The transition to medical school is a particularly challenging time for new students as they are faced with significant academic responsibilities. Moreover, for many students at the Uniformed Services University they are adjusting to being on active duty in the military. Mentoring has been considered a way to help with the transition and professional development. Prior to 2015, there was no formal mentoring program for new students at the Uniformed Services University.	Type of study: Interventional<br><br>Methodology:<br>A student-initiated program enabled preclerkship medical students to connect with residents at the Walter Reed National Military Medical Center via a secure database with resident profiles. Residents were recruited and voluntarily agreed to serve as mentors. Students were then able to access the database and reach out to mentors based on their specific goals. The program was introduced to students during orientation and student participation was completely voluntary. A survey was designed using best survey practices and sent to the 175 students participating in the program.	Medical students	The three most commonly used communication methods with mentors in precedence were in-person, e-mail, and text messages. The majority of the students found their interactions in this program<br>were beneficial and did not get in the way of their academic performance. The most common topics of discussion were academics (20%), mentor's past experience (22%), military lifestyle (7%), medical school pathways (23%), and specialty selections (23%).	• Students preferred to communicate with their mentors in person, but the flexibility of communication appears to be important.<br>• New students have a variety of academic and professional development concerns that could partially be addressed through mentoring by residents.<br>• Limitations of the program included a lack of U.S. Air Force mentors and mentors within certain residency specialties.
Sgro, Alessandro; Kamel, Mohamed Gomaa; Vuong, Thanh Huan; Ahmed,	2017	Online Research Club: A mentoring program for undergraduate students	N.A.	Type of study: Letter to Editor<br><br>Methodology: N.A.	N.A.	N.A.	• So far, our initiative has had a major role in helping undergraduate students undertake the first steps in the research field. Over the

Mahmoud, and Nguyen, Tien Huy							last five years, many outstanding young scientists managed to learn the foundations of clinical research, to the point of being able to carry out their research questions independently and to publish the results of their work in top-ranked international Journals. The contribution of students to medicine has been extraordinary and it is a potent reminder of what can be achieved by gifted and determined undergraduates undertaking a period of research
Shah, S. and Topf, J.	2019	Mentorship in the digital age: Nephrology social media collective internship	The Nephrology Social Media Collective (NSMC) internship was established in 2015 with the goal of assuring that nephrology had talented, knowledgeable people creating interesting and engaging content for the other 99%. The organizers of the NSMC internship provide opportunities and guidance in the creation of novel, nephrology-oriented free open-access medical education (FOAMed). We describe the experience from the first 4 years of the NSMC internship.	Type of study: Interventional<br><br>Methodology:<br>The organizers of the NSMC internship provide opportunities and guidance in the creation of novel, nephrology-oriented FOAMed. We describe the experience from the first 4 years of the NSMC internship.	Nephrology interns	64% rated their overall experience with the NSMC as highly valuable (5/5), 18.2% rated it as 4/5, and the remaining rated it as 3/5. Of the 22 interns that have graduated, 12 interns currently hold leadership positions in nephrology organizations and journals.	• Social media is an emerging form of collaboration that is influencing diverse areas of medicine including research, education, social support, and activism.<br>• The NSMC internship provides mentorship, teaching, and opportunities to produce creative medical education under the guidance of experienced practitioners of social media.<br>• The NSMC internship successfully deployed a worldwide education initiative and recruited a diverse cohort of

							interns who continue to generate original FOAMed content for nephrology.
Shenouda, John EA; Davies, Bethany S, and Haq, Inam	2018	The role of the smartphone in the transition from medical student to foundation trainee: a qualitative interview and focus group study	The transition from medical student to junior doctor is one of the most challenging in medicine, affecting both doctor and patient health. Opportunities to support this transition have arisen from advances in mobile technology and increased smartphone ownership.	Type of study:<br>Qualitative Review (Qualitative Analysis, Open coding, thematic analysis)<br><br>Methodology:<br>This qualitative study consisted of six in-depth interviews and two focus groups with Foundation Year 1 Trainees (intern doctors) and final year medical students within the same NHS Trust. A convenience sample of 14 participants was recruited using chain sampling. Interviews and focus groups were recorded, transcribed verbatim, analysed in accordance with thematic analysis and presented below in keeping with the standards for reporting qualitative research.	Medical Students, Junior Doctors	Participants represented both high and low intensity users. They used their smartphones to support their prescribing practices, especially antimicrobials through the MicroGuideTM app. Instant messaging, via WhatsApp, contributed to the existing bleep system, allowing coordination of both work and learning opportunities across place and time. Clinical photographs were recognised as being against regulations but there had still been occasions of use despite this. Concerns about public and colleague perceptions were important to both students and doctors, with participants describing various tactics employed to successfully integrate phone use into their practices.	• Students used their phones for learning; junior doctors used them in their clinical practice, and the feeling was that this was due to the demands of their work.<br>• Given the medico-legal risks of clinical photography and doctors' lack of awareness of the legal considerations, we recommend that students and junior doctors should avoid the use of clinical smartphone photography at present. What concerns us more is the fact that the three reported episodes were all consultant-led.
Singleton, M. H.	2016	Evaluation of a College of Medicine Peer-Mentoring Program	The purpose of the evaluation in this study was to examine whether the peer-mentoring experience was perceived as helpful to new students and how students thought the program could be improved	Type of study:<br>Sequential Mixed-method evaluation<br><br>Methodology:<br>The sequential mixed-method design consisted of a survey of 179 students and interviews of 8 students. A thematic analysis of	1st Year Medical Students	Following their participation in the program, ratings ranged from 5 (strongly agree) to 2 (disagree) that the COM Team mentoring program: made them feel part of medical school (79%), would utilize resources of support that are available (69%), found their time at medical school enjoyable (84%), were more committed to completing medical school (68%) and felt confident in succeeding in their studies (77%)	• Qualitative data revealed that students perceived the program as having had a positive impact on their confidence in succeeding in school.<br>• They felt more committed to completing school, were more likely to use resources, and report

				qualitative data was completed using a constant comparative approach.		Ratings ranged from 5 (strongly agree) to 2 (disagree) related to students' confidence: in succeeding in their studies (56%), about their academic skills (50%), in the subject knowledge (65%), and in using student services (54%)	that peer-mentoring positively affected their learning.<br>• Recommendations for program refinement include: Increased opportunities for individual mentoring, increased lunch time meetings, implement plans for initial meetings with new students prior to year one orientation, develop a systematic document for study tips, incorporate more group activities throughout the academic year, enhance the selection process for mentors with students that have similar interests and develop a curriculum for a training experience for mentors to help students prepare to meet the expectations of a physician.
Smith et al.	2015	Peer mentoring: evaluation of a new model of clinical placement in the Solomon Islands undertaken by an Australian medical school	Electives and selectives in developing countries are an important part of student learning experiences. During 2013, Bond University on the Gold Coast of Queensland in Australia piloted final year undergraduate medical student placements (n=33) at Kirakira Hospital, on Makira Island in the Solomon Islands. The placement was evaluated that year.	Type of study: Interventional study with literature review<br><br>Methodology:<br>The clinical placement in Kirakira Hospital required 12 months of planning by the Faculty of Health Sciences and Medicine of Bond University in 2012. The evaluation of 2013	Final-year medical students from Bond University Australia	Students thought the objectives of their placement had been well met.<br><br>Clinically, student respondents perceived that they could communicate well with their patients (mean 3.61), they were confident in taking a history and performing a clinical examination (mean 4.12) and they were able to suggest appropriate treatments.	• In this placement it became apparent that the student group supported one another through the challenges encountered during the placement.<br>• Students found the placement personally, professionally, environmentally, clinically and culturally enticing, as

				placements included a literature review; semi-structured interviews with participating Bond University and Kirakira Hospital staff, and with community members (n=16); an electronic survey (n=18); a focus group with participating students (n=9); and a written report with recommendations.		Professionally, respondents very much enjoyed working in a cross-cultural multidisciplinary team (mean 4.50), felt confident working in the cross-cultural environment (mean 4.28), learnt a lot about themselves during the placement (mean 4.38) and believed the work they performed was useful to the community (mean 4.28).	well as personally safe and for some a life-changing experience. It was clear that the local staff and community saw the students as making a great contribution to their healthcare needs and were keen for the program to continue.<br><br>A third area seen as important to the success of the placement is the trust built up between the community and medical program because of the continuous presence of the students across the full year.
Sutherland S. and Jalali A.	2017	Social media as an open-learning resource in medical education: current perspectives	Numerous studies evaluate the use of social media as an open-learning resource in education, but there is a little published knowledge of empirical evidence that such open-learning resources produce educative outcomes, particularly with regard to student performance.	Type of study: Systematic Review<br><br>Methodology:<br>The authors searched MEDLINE, ERIC, Embase, PubMed, Scopus, and Google Scholar from 2012 to 2017. This search included using keywords related to social media, medi- cal education, research, and evaluation, while restricting the search to peer reviewed, English language articles only. To meet inclusion criteria, manuscripts had to employ evaluative methods and undertake empirical research.	Medicine	Empirical work designed to evaluate the impact of social media as an open-learning resource in medical education is limited as only 13 studies met inclusion criteria. The majority of these studies used undergraduate medical education as the backdrop to investigate open-learning resources, such as Facebook, Twitter, and YouTube. YouTube appears to have little educational value due to the unsupervised nature of content added on a daily basis. Overall, extant reviews have demonstrated that we know a considerable amount about social media use, although to date, its impacts remain unclear.	• There is a paucity of outcome-based, empirical studies assessing the impact of social media in medical education. The few empirical studies identified tend to focus on evaluating the affective outcomes of social media and medical education as opposed to understanding any linkages between social media and performance outcomes. Given the potential for social media use in medical education, more empirical evaluative studies are required to determine educational value.

Tan et al	2016	Interprofessoinal mentorship for final-year medical students	I read with interest the article by Hawkins et al.1 on their mentorship programme for final-year medical students. Understandably, being in the final year can be stressful, and having junior doctor mentors around to provide support is certainly helpful; however, it is important not to disregard the mentorship and support that other health care professionals (e.g. nurses, pharmacists, dieticians, physiotherapists, etc.) can provide as well.	Commentary: As such, it may be worth designing and evaluating a mentorship programme where junior doctors and other experienced health care professionals are as-signed as mentors to final-year medical students, who may be partnered with other final-year health care professional students to promote interprofessional inter-action and education.2	1st Year Medical Students	N.A.	N.A.
Toh et al	2018	Toward Mentoring in Palliative Social Work: A Narrative Review of Mentoring Programs in Social Work	Variable understanding and diverse practices impede the advancement of best practices in mentoring and limit the integration of mentoring programs into undergraduate and early postgraduate programs.	Type of study: Narrative Review built on a constructivist approach and use of the Grounded theory	Medical social work involving a senior experienced mentor and a junior postgraduate and/or social work undergraduate	A total of 1302 abstracts were retrieved and evaluated, 22 full-text reviews were analysed, and 8 articles were included (Figure 1).<br><br>Seven themes were identified including<br>1. Mentoring Process<br>a. Formal and informal mentoring approaches<br>b. Initiating a mentoring relationship<br>2. Characteristics of Mentoring Relationships<br>3. The Mentor<br>4. The Mentee<br>Benefits of being mentored:<br>a. Professional socialization<br>b. Research<br>c. Career progressions (short- and long-term goals)<br>d. Personal support<br>5. Organisation and Stakeholders<br>6. Barriers to Mentoring<br>7. Mentoring Outcomes	• Mentoring in medical social work offers a unique and effective means of providing medical social workers with holistic and individualized support through the establishment of effective mentoring relationships. Success of this process pivots upon personal relationships and emphasizes once more the individualized nature of the process. This relational element of mentoring relies upon the presence of a structured and organisationally supported mentoring programs to promote timely, consistent, appropriate, personalized, and

							flexible support of mentees and mentors often in evolving conditions.<br>- Sustaining such a program demands require regular evaluation of the effectiveness and efficiency mentoring processes, individual mentoring relationships and their outcomes, the impact of mentoring upon patient care and mentee and mentor welfare, research output, and robust studies of the key aspects of the mentoring process.
Underhill et al	2010	Mentoring in the management of hematological malignancies	The Mentoring in Management of Haematological Malignancies (MMHM) project aimed to improve treatment outcomes, coordinate care and provide best practice for patients with haematological cancers, by developing a program of mentoring and multidisciplinary care between a regional and a metropolitan centre.	Type of study: Interventional with qualitative feedback<br><br>Methodology:<br>A regular multidisciplinary meeting conducted by teleconference was established between a tertiary metropolitan site and a regional practice to discuss cases of patients with hematological malignancies. Information from multidisciplinary team meetings was recorded to capture adherence to process and clinician outcomes. An educational program was developed. A gap analysis was performed to identify differences in routine	Multidisciplinary carers of hematological cancer patients	N.A.	• This project demonstrated some improvements in the development of multidisciplinary care for regional hematology patients it was hampered by a lack of financial support for the pathology review process.<br>• The process is also reliant on an administrator who is able to track samples in the system and intervene when appropriate.<br>• The technology to review radiology images online is still an indemnity issue.<br>• Additional concerns raised included

				practice between the two centers. Clinician satisfaction with mentoring and educational interventions were assessed by structured survey.			difficulty with taking on extra work with an already heavy clinical load. There is a need to develop further models of support to facilitate and support the review of pathology specimens for the management of cancers that are complex or low in volume.<br>•
Vogelsang, Markus; Rockenbauch, Katrin; Wrigge, Hermann; Heinke, Wolfgang, and Hempel, Gunther	2018	Medical Education for "Generation Z": Everything online?! – An analysis of Internet-based media use by teachers in medicine	The aims of this study were to gain an overview of the web-based media used during the clinical phase of medical study at German medical schools and to identify the resources needed for web-based media use. Also examined were the influences on web-based media use, for instance, the assessment of their suitability for use in teaching.	Type of study: Quantitative Study<br><br>Methodology:<br>An online survey of 264 teacher coordinators in internal medicine, surgery, anesthesiology, gynecology, pediatrics and psychiatry was conducted in March and April, 2016. This survey was carried out in the German-speaking countries using a 181-item questionnaire developed by us. Analysis took place in the form of descriptive and exploratory data analysis	Medical students	The response rate was 34.8% with 92 responses. Individual web-based media were actively used in the classroom by a maximum of 28% of participants. Reasons cited against using web-based media in teaching included the amount of time required and lack of support staff. The assessment of suitability revealed that interactive patient cases, podcasts and subject-specific apps for teaching medicine were predominantly viewed as constructive teaching tools. Social media such as Facebook and Twitter were considered unsuitable. When using webbased media and assessing their suitability for teaching, no correlations with the personal profiles of the teachers were found in the exploratory analysis, except regarding the use of different sources of information.	• The assessment of suitability revealed that interactive patient cases, podcasts and subject-specific apps for teaching medicine were predominantly viewed as constructive teaching tools. Social media such as Facebook and Twitter were considered unsuitable. When using web-based media and assessing their suitability for teaching, no correlations with the personal profiles of the teachers were found in the exploratory analysis, except regarding the use of different sources of information. Despite the Internet's rapid development in the past 15 years, web-based media continue to play only a minor role in teaching

							medicine. Above all, teacher motivation and sufficient staff resources are necessary for more effective use of Internet-based media in the future.<br>•
Walsh, K	2015	Online mentoring in medical education	To outline the advantages and disadvantages of online mentoring and suggest ways of maximising the advantages and minimising the disadvantages	Type of study: Editorial	Unspecific	N.A.	• Mentoring is different from supervision, teaching and assessment. Mentoring should be formative and confidential relationship<br>• The advantages of online mentoring is that it is more mentee centric, less expensive and allows for a more continuous and long-term relationship than traditional forms of mentoring.<br>• The criticisms of online mentoring is that it hinders non-verbal communication, there may be less trust between the mentor and mentee and that a reliable and consistent internet connection is required
Wahab et al	2016	Creating Effective Interprofessional Mentoring Relationships in Palliative Care- Lessons from Medicine, Nursing, Surgery and Social Work	Wahab et al (2016) suggest that IPM be “delivered by intentionally created, usually relatively small work groups in health care [1,3], who are recognized by others as well as by themselves as having a collective identity and shared responsibility for a patient or group of patients” This team “must display “the levels of cooperation, coordination and collaboration characterizing the relationships between professions in delivering patient-centered care”	Methodology:<br>Built on a constructivist approach and use of the Grounded theory	Undergraduate and postgraduate medicine, surgery, nursing and social work	1059 abstracts were retrieved and evaluated, 61 full-text reviews were reviewed, 20 reviews were included, and thematic analysis revealed 6 themes including: (1) characteristics of prevailing definitions of mentoring, (2) characteristics of mentoring relationships, (3) characteristics of mentors and (4) mentees, (5) benefits of mentoring, (6) drawbacks of mentoring and finally how they all tie into painting a preferred mentoring partnership.	• Conceptual analysis suggests that IPE is built upon elements of multiple learning theories such as constructivism (learning through experiences and discussion), cognitivism (organisation of information via cognitive processes),

							humanism (learning by self-actualisation) and cognitive apprenticeship
Warren et al	2012	Use of an innovative web-based mentoring tool to guide residents and faculty in design of original clinical research	CoolResearcher.com: a web-based mentoring tool for assisting faculty, resident, and student novices, providing stepwise guidance with developing the project. Initiating a research project is often a difficult and intimidating task for a newer faculty member, resident, or student who does not have prior experience, thus necessitating the use of a mentor.	Methodology: This innovation is an online tool which provides the first two steps of that process, a site which leads the user through the initial steps of research design. The user is able to present an idea to the mentor with some degree of basic development and sophistication, thus making greater use of the mentor's time. When complete, the user sends the resultant design entered into blank fields via automated e-mail to the mentor prior to a live meeting to discuss the results and further progress.	Faculty, resident, and student novices	N.A.	• Henri (1992) described mentoring as a three-step process, the first of which involves communication from the mentor providing the starting information; second the student/mentee responds to this information, and third, there is a reply.
Wright et al	2019	Training the next generation of clinical rheumatology researchers: Evaluation of a graduate allied health professional and nurse internship programme	Building research capacity is an essential part of sustaining evidence-based practice in nursing and allied health professions working within rheumatology. Whilst medical and dental professions have a strong tradition of research capacity building, the situation for allied health Professionals and nursing in the UK is less developed. This presentation reports on the interim findings of an evaluation of a collaborative internship programme across five UK universities from 2015-18. The internship included an eight-week programme of structured training workshops and research project delivery, and ongoing	Type of study: Quantitative analysis<br><br>Methodology: The evaluation employed mixed methods including: analysis of research metrics, an annual evaluation questionnaire sent to all 16 interns, and qualitative email interviews (8 interns, 9 mentors) conducted at the end of the internship programme. Interpretive	Clinical rheumatology researchers	Skills attained of most value to interns were technical research (e.g. qualitative research), research process (e.g. securing funding), rheumatology knowledge (e.g. fatigue), and general skills (e.g. communication). Two domains of impact were identified. First, the programme directly impacted on research careers with four interns securing clinical academic positions and most others reporting commitment to pursuing active research in the near future. Second, the internship had an impact on practice for those entering full-time clinical careers. Interns spoke of their ability to be 'critically aware', seeking an evidence base for clinical decisions.	• Many spoke of a new confidence in expressing opinions with clinical colleagues.<br>• Others spoke of the need for patient-centered care, learned from the Patient and Public Involvement training provided by the internship.<br>• Similarly, interns reported an increased awareness of the wider relevance of rheumatology, which influenced their

			mentoring by experienced researchers. Sixteen interns were recruited from across the UK, including physiotherapists (7), podiatrists (5), occupational therapists (2) and nurses (2).	phenomenological analysis of transcripts was used to identify recurring themes.			subsequent clinical practice.<br>• One challenge for the internship programme identified by mentors was the difficulty in attracting interns from all Allied Health Professionals and Nursing backgrounds (notably nurses). In addition, several interns entering fulltime clinical roles reported difficulties in continuing research in environments that devalued such activity.
Wu et al	2016	Toward an Interprofessional Mentoring Program in Palliative Care - A Review of Undergraduate and Postgraduate Mentoring in Medicine, Nursing, Surgery and Social Work	Wahab et al (2016) identified IPM as a means of achieving PM's care goals and inculcate Interprofessional education and carried a review of IPM in the presence of a wide array of mentoring practices and the fast evolving nature of mentoring approaches.	Methodology:<br>Built on a constructivist approach using the Grounded approach	Undergraduate and Postgraduate Medicine, Nursing, Surgery and Social Work involving senior clinicians and junior doctors and/or medical students	1059 abstracts were retrieved and evaluated, 61 full-text reviews were reviewed, 20 reviews were included, and 3 themes were identified (1) Definition – dynamic, mutually beneficial, context dependent, goal specific process involving knowledge transfer from a more-experienced mentor to a less-experienced mentee (2) Components of mentoring- formal and informal mentoring, Developmental Process- includes relational characteristics, group, mosaic, dyadic, e-mentoring (3) elements of the mentoring process – mentor training, duration and frequency	• There is consistency between UG and PG mentoring.<br>• Formal program<br>- provides mentors with financial resources and protected time that enable them to better respond to the needs of mentees.<br>- Facilitate an effective matching process.<br>- provide technological support for long distance mentoring.<br>- provide guidance for mentees as they select an appropriate mentor, in meeting their responsibilities and navigating their respective roles within their mentoring relationships.<br>- provide for standardized training and regular refreshers

							for mentors on mentoring, communications and support skills<br>- support interprofessional education and facilitate interprofessional teamwork.<br>• IPM facilitates Interprofessional Education through<br>- Didactic lectures remain critical to provide systematic, core knowledge , focusing on roles of different healthcare professionals in Palliative Care role of interprofessional collaboration as well as concepts of teamwork and collaboration<br>- Simulation-based learning aligns with elements of experiential learning integral in IPE developing team communication and collaboration<br>- Simulations also promote observation-based learning as those not currently involved can observe the roles of their colleagues and reflect<br>- Case discussions include either discussions of cases from clinical practice or developed as curriculum content. These discussions aim to reinforce understanding of

							interprofessional collaboration taught in didactic lectures exchanged-based learning and action-based learning through collaborative enquiry.<br>- Clinical rotations can be modeled after the Exemplary Care and Learning Sites (ECLS) model. The model promotes interprofessional clinical learning by integrating mentees into interprofessional teams and allowing them to actively participate in clinical improvement efforts. This facilitates both practice-based and observational learning.<br>• Supporting this is a e-mentoring program
Yap et al	2017	Thematic Review of Mentoring in Occupational Therapy and Physiotherapy between 2000 and 2015, Sitting Occupational Therapy and Physiotherapy in A Holistic Palliative Medicine Multidisciplinary Mentoring Program.	Mentoring is seen as an effective means of improving skills, attitudes and practices in caring for dying patients", providing holistic and personalized support and advancing the academic and research interests amongst trainees. However, the absence of a clear definition and the presence of diverse mentoring practices limits understanding and use of mentoring in PM. This review is aimed at filling the gap in mentoring knowledge in OT and PT. Concurrent evaluation of mentoring programs in OT and PT is justified given that both specialties will be used together in MDT mentoring programs.	Type of study: Thematic Review with Braun and Clarke's approach	Occupational therapy and physiotherapy in Palliative medicine	3407 abstracts on OT were retrieved, 44 full-text articles were analyzed and 5 papers were included in this review.<br><br>Separate thematic analysis of PT and OT papers revealed 3 themes including (1) defining mentoring, (2) benefits of mentoring and (3) mentors and mentees' perspective about the mentoring process.	• OT and PT mentoring is relational-, organisational-, mentee-, mentor-dependent and is evolving.<br>• Consistent, timely, holistic and individualized support that cuts across specialties provided by senior members of the MDT in a structured program that will provide transparency and accessibility to mentoring in PM is required. This is particularly evident in the knowledge that PM can no longer afford to

							train its trainees along specialty lines.<br>• This review suggests that inclusion of OT and PT into a MDT mentoring program is possible but does raise questions as to how best to approach the design of a consistent mentoring approach.
